# A Secreted NlpC/P60 Endopeptidase from Photobacterium damselae subsp. *piscicida* Cleaves the Peptidoglycan of Potentially Competing Bacteria

**DOI:** 10.1128/mSphere.00736-20

**Published:** 2021-02-03

**Authors:** Johnny Lisboa, Cassilda Pereira, Aline Rifflet, Juan Ayala, Mateus S. Terceti, Alba V. Barca, Inês Rodrigues, Pedro José Barbosa Pereira, Carlos R. Osorio, Francisco García-del Portillo, Ivo Gomperts Boneca, Ana do Vale, Nuno M. S. dos Santos

**Affiliations:** a Fish Immunology and Vaccinology Group, Instituto de Biologia Molecular e Celular (IBMC), Universidade do Porto, Porto, Portugal; b Fish Immunology and Vaccinology Group, Instituto de Investigação e Inovação em Saúde (i3S), Universidade do Porto, Porto, Portugal; c Institut Pasteur, Unité Biologie et Génétique de la Paroi Bactérienne, Paris, France; d INSERM Groupe Avenir, Paris, France; e CNRS, UMR “Integrated and Molecular Microbiology,” Paris, France; f Centro de Biología Molecular Severo Ochoa (CBMSO), Consejo Superior de Investigaciones Científicas (CSIC), Madrid, Spain; g Departamento de Microbioloxía e Parasitoloxía, Instituto de Acuicultura, Universidade de Santiago de Compostela, Santiago de Compostela, Spain; h Biomolecular Structure Group, Instituto de Biologia Molecular e Celular (IBMC), Universidade do Porto, Porto, Portugal; i Macromolecular Structure Group, Instituto de Investigação e Inovação em Saúde (i3S), Universidade do Porto, Porto, Portugal; j Laboratorio de Patógenos Bacterianos Intracelulares, Centro Nacional de Biotecnología (CNB), Consejo Superior de Investigaciones Científicas (CSIC), Madrid, Spain; Escola Paulista de Medicina/Universidade Federal de São Paulo

**Keywords:** NlpC/P60, *Vibrio anguillarum*, *Vibrio vulnificus*, X-ray crystallography, cell wall hydrolases, peptidoglycan, *Photobacterium damselae* subsp. *piscicida*, type II secretion system

## Abstract

Peptidoglycan (PG) is a major component of the bacterial cell wall, forming a mesh-like structure enwrapping the bacteria that is essential for maintaining structural integrity and providing support for anchoring other components of the cell envelope. PG biogenesis is highly dynamic and requires multiple enzymes, including several hydrolases that cleave glycosidic or amide bonds in the PG. This work describes the structural and functional characterization of an NlpC/P60-containing peptidase from Photobacterium damselae subsp. *piscicida* (*Phdp*), a Gram-negative bacterium that causes high mortality of warm-water marine fish with great impact for the aquaculture industry. PnpA (*Photobacterium* NlpC-like protein A) has a four-domain structure with a hydrophobic and narrow access to the catalytic center and specificity for the γ-d-glutamyl-*meso*-diaminopimelic acid bond. However, PnpA does not cleave the PG of *Phdp* or PG of several Gram-negative and Gram-positive bacterial species. Interestingly, it is secreted by the *Phdp* type II secretion system and degrades the PG of Vibrio anguillarum and Vibrio vulnificus. This suggests that PnpA is used by *Phdp* to gain an advantage over bacteria that compete for the same resources or to obtain nutrients in nutrient-scarce environments. Comparison of the muropeptide composition of PG susceptible and resistant to the catalytic activity of PnpA showed that the global content of muropeptides is similar, suggesting that susceptibility to PnpA is determined by the three-dimensional organization of the muropeptides in the PG.

**IMPORTANCE** Peptidoglycan (PG) is a major component of the bacterial cell wall formed by long chains of two alternating sugars interconnected by short peptides, generating a mesh-like structure that enwraps the bacterial cell. Although PG provides structural integrity and support for anchoring other components of the cell envelope, it is constantly being remodeled through the action of specific enzymes that cleave or join its components. Here, it is shown that Photobacterium damselae subsp. *piscicida*, a bacterium that causes high mortality in warm-water marine fish, produces PnpA, an enzyme that is secreted into the environment and is able to cleave the PG of potentially competing bacteria, either to gain a competitive advantage and/or to obtain nutrients. The specificity of PnpA for the PG of some bacteria and its inability to cleave others may be explained by differences in the structure of the PG mesh and not by different muropeptide composition.

## Abstract (precis)

Peptidoglycan (PG) is a major component of the bacterial cell wall formed by long chains of two alternating sugars interconnected by short peptides, generating a mesh-like structure that enwraps the bacterial cell. Although PG provides structural integrity and support for anchoring other components of the cell envelope, it is constantly being remodeled through the action of specific enzymes that cleave or join its components.

## INTRODUCTION

Peptidoglycan (PG) is a major component of the bacterial cell wall, essential for maintaining structural integrity and internal osmotic pressure, shaping the morphology of bacteria, and providing support for anchoring other components of the cell envelope (1, 2). PG forms a mesh-like structure that enwraps the bacterial cell, referred to as sacculus, which is composed of long chains of two alternating β(1-4) glycosidic-bonded glycans, *N-*acetylglucosamine (GlcNAc) and *N*-acetylmuramic acid (MurNAc), cross-linked by short stem peptides, either directly or through bridging peptides (1, 3–5). The stem peptides are usually 4 or 5 amino acids long, contain l- and d-amino acids, and extend from MurNAc (1–4). The most common structure of the stem peptide is l-Ala-γ-d-Glu-mDAP-d-Ala-d-Ala (mDAP stands for *meso*-diaminopimelic acid) in Gram-negative bacteria and l-Ala-γ-d-Glu-l-Lys-d-Ala-d-Ala in Gram-positive organisms (1, 2, 4).

In spite of its stabilizing function, PG is highly dynamic, with covalent bonds being formed and broken by different enzymes. Multiple hydrolases, capable of cleaving glycosidic (glycosidases) or amide (amidases and peptidases) bonds in the PG sacculus and/or its soluble fragments, play a preponderant role in PG dynamics (1, 2, 6–13). Degradation products resulting from the catalytic activity of PG hydrolases can be recycled for PG *de novo* biosynthesis and also act as signaling molecules in quorum sensing, triggering antibiotic resistance or regrowth of dormant cells or as effector molecules in immune responses (1, 2, 6, 7, 12, 14, 15). Besides their role in PG dynamics, hydrolases can also be secreted to the environment or injected via type VI secretion systems into the periplasm of other bacteria to confer competitive advantage over competing bacteria that share mixed growth environments or as a way of obtaining nutrients (1, 10, 11, 16–23).

PG peptidases are a widely diverse group of enzymes, with 10 different types of catalytic domains involved in PG hydrolysis described thus far (1, 24). Of these enzymes, cysteine peptidases containing new lipoprotein C/protein of 60-kDa (NlpC/P60) catalytic domains are present in most bacterial lineages, suggesting that they play an important biological role (1, 24). NlpC/P60-containing peptidases are involved in the catalysis of the *N*-acetylmuramate-l-alanine or d-γ-glutamyl-*meso*-diaminopimelate linkages, with four major groups identified so far: (i) P60-like, (ii) AcmB/LytN-like, (iii) YaeF/poxvirus G6R, and (iv) lecithin retinol acyltransferase (LRAT)-like (24). The NlpC/P60 domain is structurally similar to a primitive papain-like peptidase (24–29) and can be found alone or fused to other domains, with or without catalytic functions, to form multifunctional proteins (1, 2, 24, 26, 30–35). Several of these domains, such as the SH3 (sarcoma homology 3) domain (31, 32, 35), are involved in anchoring hydrolases to cell wall components, allowing their appropriate concentration and positioning for the formation of an efficient enzyme-substrate complex (1).

Photobacterium damselae subsp. *piscicida* (*Phdp*) is a Gram-negative, halophilic bacterium that induces an acute infection that rapidly develops into septicemia, resulting in high mortality of warm-water marine fish with devastating consequences for the aquaculture industry (36, 37). Although it has been suggested that *Phdp* remains in a cultivable form in salt water for only 4 or 5 days (38, 39), it was also suggested that it has the ability to enter a dormant, noncultivable but infectious state in salt water and sediment (40). With regard to the mechanisms responsible for the pathogenicity of *Phdp*, it was shown that extracellular products (ECPs) play a fundamental role (41, 42) although among their components, only the toxin AIP56 has been identified and characterized so far (43–47).

The present work reports the structural and functional characterization of a novel NlpC/P60-containing peptidase from *Phdp* (PnpA). The results show that PnpA is a PG hydrolase with a four-domain structure similar to that of Desulfovibrio vulgaris lysin (DvLysin) and specificity for the γ-d-glutamyl-*meso*-diaminopimelic acid bond (26), but with a more hydrophobic and narrower access to the catalytic center. It is also shown that PnpA is secreted into the extracellular medium by the *Phdp* type II secretion system and acts on the PG of Vibrio anguillarum and Vibrio vulnificus, suggesting that it may provide *Phdp* an advantage over bacteria competing for the same resources or a way of obtaining nutrients in nutrient-scarce environments, either inside or outside the host. Comparison of the muropeptide compositions of PG, susceptible and resistant to PnpA activity, allowed development of a model suggesting that the susceptibility to PnpA is determined by three-dimensional structural features of the PG and not by their chemical compositions.

## RESULTS

### Photobacterium damselae subsp. *piscicida* secretes an NlpC/P60 family protein.

Photobacterium damselae subsp. *piscicida* (*Phdp*) virulent strains have a relatively simple profile of secreted proteins in mid-exponential-growth-phase cultures (45). Apart from AIP56 toxin, no other proteins have been identified and characterized. Sodium dodecyl sulfate-polyacrylamide gel electrophoresis (SDS-PAGE) analysis of proteins from *Phdp* extracellular products (ECPs) precipitated with trichloroacetic acid (TCA) revealed a band of approximately 55 kDa that was excised from the gel and subjected to matrix-assisted laser desorption ionization−time of flight mass spectrometry (MALDI-TOF MS). The obtained MS data were used in a Mascot search against the NCBI database resulting in the identification of a hypothetical protein from Photobacterium damselae subsp. damselae (*Phdd*) CIP 102761 (VDA_000779; NCBI accession number EEZ39759). The 1,479-nucleotide homologous sequence in the *Phdp* MT1415 strain (accession number TJZ86030.1) was then amplified using primers designed based on the VDA_000779 sequence. *In silico* analysis (SignalP 5.0 and NCBI conserved domain search) of its 499-amino-acid translation product predicted a Sec signal peptide (M^1^ to A^19^), followed by an N_NLPC_P60 putative stabilizing domain (Pfam PF12912), an SH3b1 (Pfam PF12913/12914), and an NlpC_P60 domain (Pfam PF00877), classifying it as a protein belonging to the NlpC/P60 family, hereafter referred to as PnpA (*Photobacterium* NlpC-like protein A).

### PnpA is encoded in a genetically unstable chromosomal region, and its expression levels are similar at exponential and stationary phases of growth.

**FIG 1:**
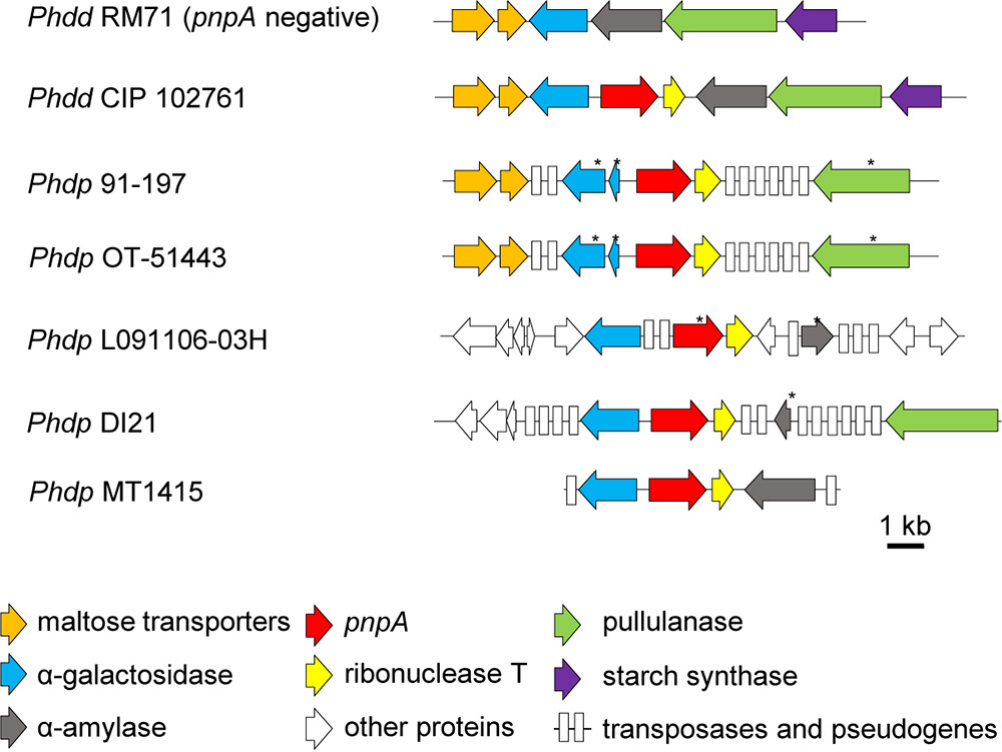
Genomic context of *pnpA*. Schematic representation of the genomic context of *pnpA* (shown in red) in the indicated Photobacterium damselae subsp. damselae (*Phdd*) and Photobacterium damselae subsp. *piscicida* (*Phdp*) strains. An asterisk denotes a truncated gene version.

To investigate the genetic context of *pnpA* in *Phdp* MT1415 strain, the draft genome sequence of MT1415 was obtained in this study. Then, homologous DNA sequences of a number of *Phdp* and *Phdd* isolates were additionally retrieved from the GenBank database and subjected to comparative sequence analysis (Fig. 1). This revealed that the PnpA-encoding gene is invariably linked to a downstream gene encoding an RNase T and to an upstream gene encoding an α-galactosidase, the latter being a pseudogene in some *Phdp* isolates. As a whole, the DNA flanking *pnpA* underwent a massive insertion of transposase genes (IS elements of the IS*1* and IS*91* families) likely followed by accumulation of inactivating mutations, resulting in a collection of pseudogenes. This process of gene decay not only affected the transposase genes themselves but also flanking genes encoding enzymes putatively involved in sugar metabolism, as α-galactosidases, α-amylases, and pullulanases (Fig. 1). Proliferation of insertion sequences that cause a high frequency of pseudogenes and gene loss is indeed a hallmark of all *Phdp* genomes studied thus far (48–50). The observation that PnpA- and the RNase T-encoding genes have escaped the inactivation by IS insertions suggests that these two genes may fulfill an important role in *Phdp*.

Expression levels of *pnpA* were determined by reverse transcription-PCR (RT-PCR), showing that under the culture conditions used (growth in tryptic soy broth supplemented with NaCl to a final concentration of 1% [wt/vol] [TSB-1] at 25°C), there are no differences in the level of gene transcription between exponential- and stationary-phase cultures (see Fig. S1 in the supplemental material).

10.1128/mSphere.00736-20.3 FIG S1 *In vitro* expression of *pnpA*. Expression levels in Photobacterium damselae subsp. *piscicida* MT1415 strain at exponential (OD_600_ of 0.4) and stationary (OD_600_ of 1.2) phases were determined using quantitative RT-PCR, normalized based on expression of the housekeeping gene 16S and expressed as 2^−ΔΔCT^. Data represent the means ± SD of three independent experiments with all measurements done in triplicate. Statistical significance between expression at exponential and stationary phase was assessed using Student’s *t* test. ns, not significant. Download FIG S1, TIF file, 0.1 MB. Copyright © 2021 Lisboa et al. 2021 Lisboa et al. This content is distributed under the terms of the Creative Commons Attribution 4.0 International license.

### Overall description of PnpA structure.

For better understanding of the structure-function relationship of PnpA, its three-dimensional structure was solved. The crystal structure of PnpA was determined at 1.4-Å resolution by molecular replacement with DvLysin (PDB entry 3M1U, 26% sequence identity), an endopeptidase from Desulfovibrio vulgaris Hildenborough (26). The crystal asymmetric unit contains two PnpA molecules, which are essentially identical (root mean square deviation [RMSD], of 0.5 Å for 457 aligned Cα atoms). Table S1 in the supplemental material summarizes the data collection, processing, and refinement statistics.

10.1128/mSphere.00736-20.1 TABLE S1 Data collection and refinement statistics of PnpA. The numbers in parentheses are for the highest resolution shell. Download Table S1, PDF file, 0.1 MB. Copyright © 2021 Lisboa et al. 2021 Lisboa et al. This content is distributed under the terms of the Creative Commons Attribution 4.0 International license.

**FIG 2:**
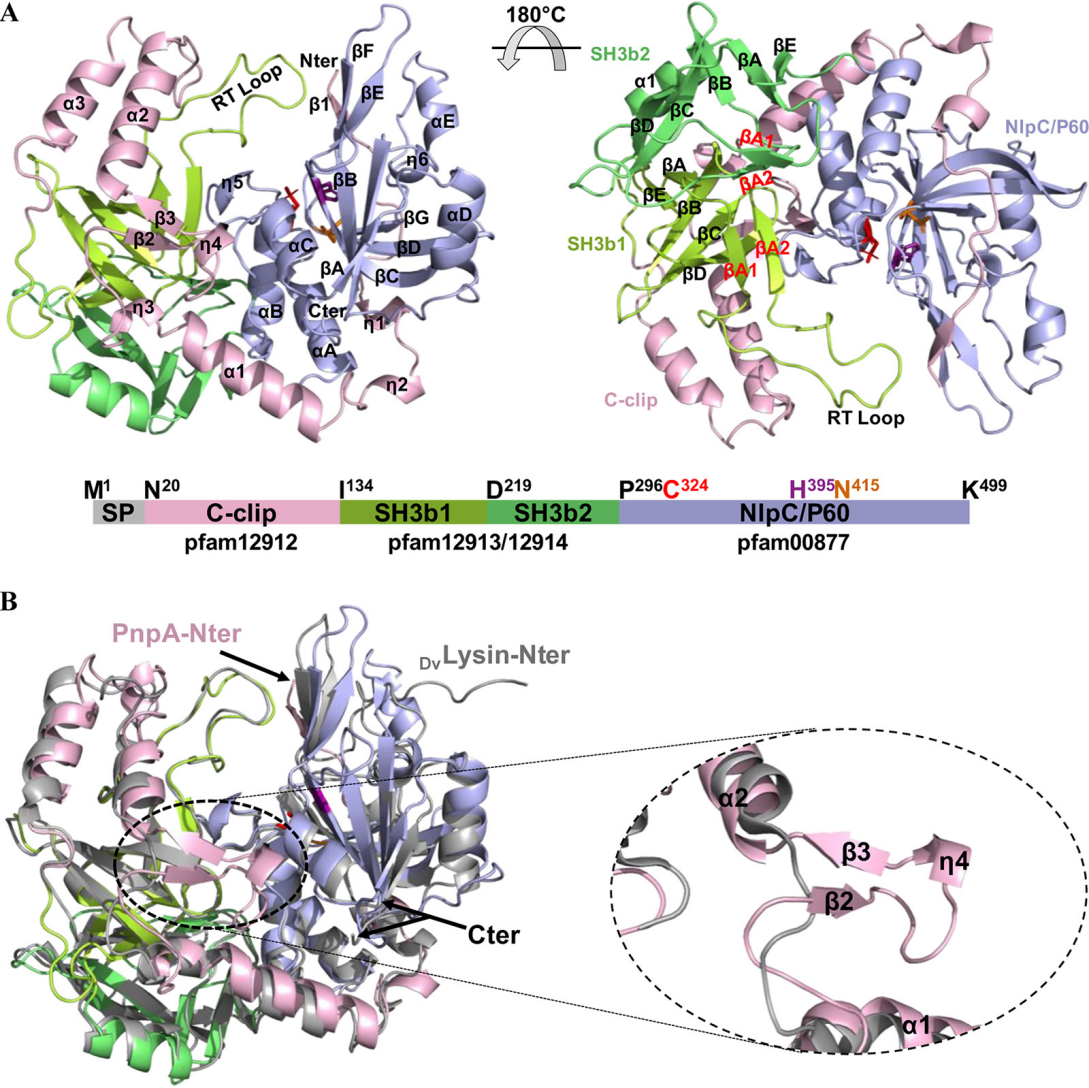
Three-dimensional structure of *Phdp* PnpA. (A) Cartoon representation of the PnpA monomer, with domains colored as in the linear representation shown below. The signal peptide (SP) (gray), C-clip domain (pink), SH3b1 domain (light green), SH3b2 domain (dark green), NlpC/P60 domain (purple), and domain boundaries and catalytic residues are indicated. The catalytic site residues are represented as sticks (C324 [red], H395 [magenta], and N415 [orange]). The N and C termini (Nter and Cter, respectively) and secondary structure elements are labeled. (B) Cartoon representation of superposed PnpA (color code as in panel A) and DvLysin (gray). N and C termini are indicated. A close-up of the insertion between α1 and α2, forming an additional antiparallel β-sheet (β2 and β3) and a 3_10_ helix (η4) in the c-clip domain, is shown in the insert (dashed oval).

Analysis of the intermolecular packing interfaces within the crystal lattice suggests that the molecule behaves as a monomer in solution, which is in agreement with the molecular mass estimated by size exclusion chromatography. The PnpA monomer has an overall structure similar to that of DvLysin (26), namely, one N-terminal “c-clip” or “N_NLPC_P60” stabilizing domain (residues N^20^-N^133^), two SH3b domains (SH3b1, residues I^134^-V^218^; SH3b2, residues D^219^-T^295^), and the C-terminal NlpC/P60 catalytic domain (residues P^296^-K^499^) (Fig. 2A). The three-dimensional models of DvLysin and PnpA display an RMSD of 2.2 Å (for 405 aligned Cα atoms), suggesting that both proteins may be functionally equivalent. A significant number of structures sharing at least one of the PnpA domains have been identified (Table S2), although so far, PnpA and DvLysin are the only four-domain NlpC/P60-containing peptidases whose structure has been reported.

10.1128/mSphere.00736-20.2 TABLE S2 Structural comparison of PnpA and other bacterial proteins with at least one common domain. Alignments were performed using the DALI structural comparison server (L. Holm, Bioinformatics 35:5326−5327, 2019, https://doi.org/10.1093/bioinformatics/btz536; L. Holm, Methods Mol Biol 2112:29−42, 2020, https://doi.org/10.1007/978-1-0716-0270-6_3) with full-length PnpA as a search probe. For proteins with multiple SH3-like domains or multiple chains, only the best match is shown. Footnotes: †, number of residues present in the model used for comparison; ‡, Joint Center for Structural Genomics; §, Northeast Structural Genomics Consortium; δ, Ontario Centre for Structural Proteomics; a, Z-score is a measure of the quality of the alignment. The higher the Z-score, the more homologous are the structures. Download Table S2, PDF file, 0.2 MB. Copyright © 2021 Lisboa et al. 2021 Lisboa et al. This content is distributed under the terms of the Creative Commons Attribution 4.0 International license.

As in DvLysin (26), the PnpA c-clip domain has an extended helical conformation which surrounds and stabilizes the SH3b1 and NlpC/P60 domains, forming a planar assembly from which the SH3b2 domain protrudes (Fig. S2). Compared to DvLysin, the c-clip domain of PnpA harbors an extension between helices α1 and α2, thereby forming an additional two-stranded antiparallel β-sheet (β2 and β3) and a 3_10_ helix (η4), which protrude into the catalytic groove and close one of its sides (Fig. 2B).

10.1128/mSphere.00736-20.4 FIG S2 Cartoon representation of the three-dimensional structure of PnpA highlighting the protrusion of the SH3b2 domain relative to the plane defined by the other domains. Color code is as shown in Fig. 2. Download FIG S2, TIF file, 1.3 MB. Copyright © 2021 Lisboa et al. 2021 Lisboa et al. This content is distributed under the terms of the Creative Commons Attribution 4.0 International license.

The presence of SH3b domains in prokaryotes has long been documented. These domains have been described as targeting domains, involved in cell wall recognition and binding (1, 24, 35). Despite the lack of amino acid sequence conservation (8% sequence identity), the two SH3b domains in PnpA have a conserved overall fold (RMSD of 3.9 Å for 55 aligned Cα atoms) (Fig. S3). As in DvLysin (26), both PnpA SH3b domains consist of seven conserved strands (βA-βA1-βA2-βB-βC-βD-βE), with the βA-βE strands structurally equivalent to their eukaryotic counterparts (31, 32), while βA1 and βA2 form a β-hairpin that corresponds to the RT loops of eukaryotic SH3b domains (Fig. 2A).

10.1128/mSphere.00736-20.5 FIG S3 Structural comparison of the SH3b domains of PnpA and DvLysin (PDB entry 31MU). (A) Superposition of SH3b1 (light green) and SH3b2 (dark green) domains from PnpA (left), of SH3b1 domains from PnpA (light green) and DvLysin (gray) (middle), and of SH3b2 domains from PnpA (dark green) and DvLysin (gray) (right). (B) Results of the pairwise superposition of SH3b domains using the DALI server (L. Holm, Bioinformatics 35:5326−5327, 2019, https://doi.org/10.1093/bioinformatics/btz536; L. Holm, Methods Mol Biol 2112:29−42, 2020, https://doi.org/10.1007/978-1-0716-0270-6_3). Footnote a, Z-score is a measure of the quality of the alignment. The higher the Z-score, the more homologous are the structures. Download FIG S3, TIF file, 0.6 MB. Copyright © 2021 Lisboa et al. 2021 Lisboa et al. This content is distributed under the terms of the Creative Commons Attribution 4.0 International license.

As in other NlpC/P60-containing peptidases, the 204-residue-long C-terminal NlpC/P60 catalytic domain of PnpA displays a fold resembling a primitive papain-like cysteine peptidase (24). Its secondary structure elements adopt the topology described for DvLysin, i.e., a six-stranded central β-sheet and five α-helices with αA-αB-αC-βA-αD-βB-βC-βD-βE-αE-βF topology, where αA-αB-αC and αD-αE protect either side of the central β-sheet (Fig. 2A) (26).

### PnpA has a narrow and hydrophobic access to the catalytic site.

**FIG 3:**
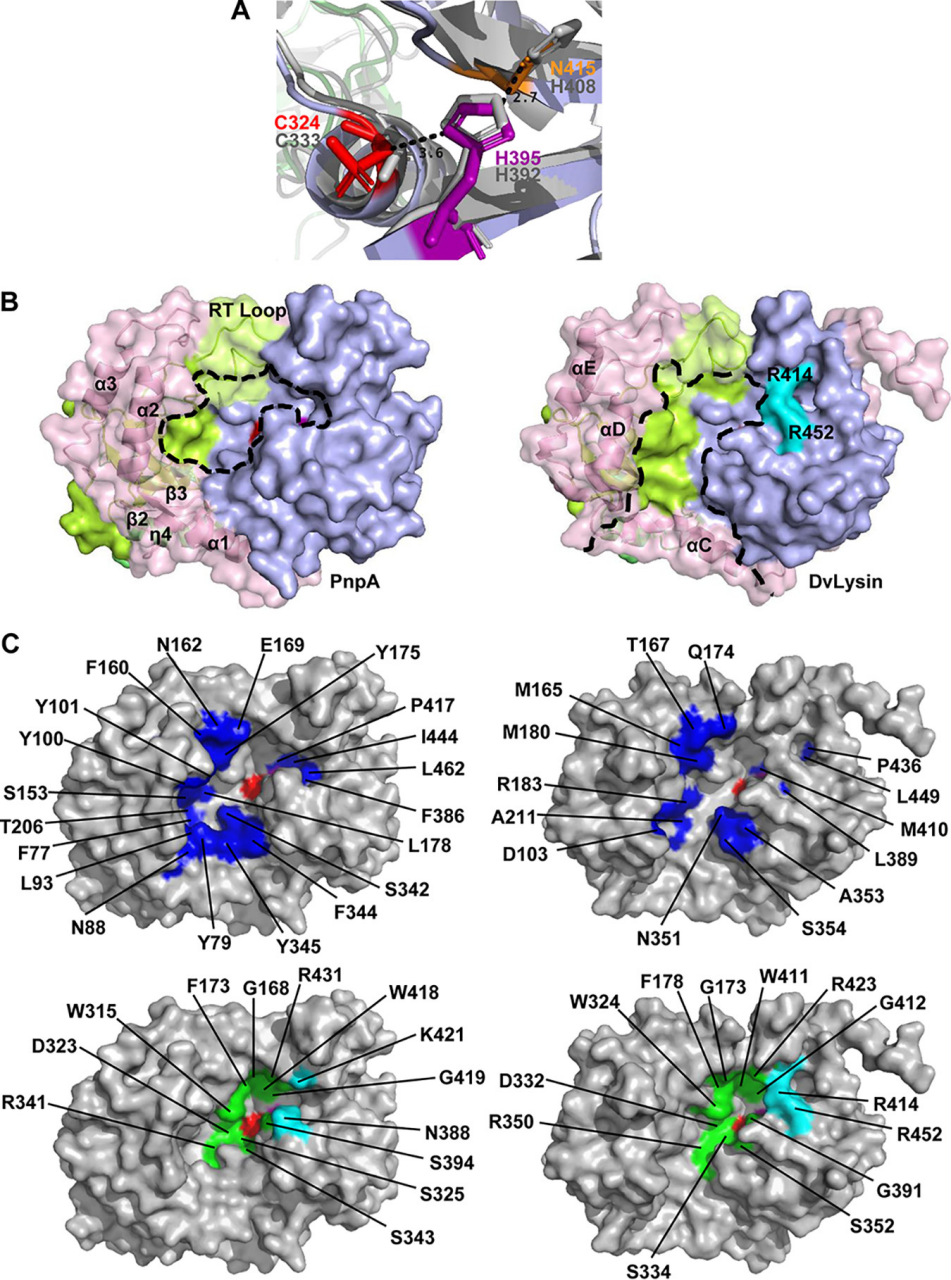
Structural comparison of the active sites of PnpA and DvLysin. (A) Superposition of the catalytic site of PnpA (colored sticks) and DvLysin (gray sticks). Dashed line indicates the distance between amino acid residues in angstroms. (B) Solid surface representation of PnpA (left) and DvLysin (right). Catalytic grooves are outlined by dashed lines. Residues R414 and R452 from DvLysin are colored cyan and labeled. (C) Comparison of the catalytic cavities of PnpA (left) and DvLysin (right). Hydrophobic and polar residues close to the substrate binding region are colored dark blue (top panel). DvLysin residues involved in substrate binding (27) and conserved in PnpA are colored green (bottom panel). Catalytic residues are colored as in Fig. 2A.

The active site of NlpC/P60 cysteine peptidases consists of a conserved cysteine-histidine dyad and a third polar residue (H, N, or Q) that orients and polarizes the catalytic histidine (24–29). In PnpA, the residues that make up the active site are C324, H395, and N415, the latter similar to the equivalent residue found in the active site of the prototypical papain (51), but differing from the histidine (H408) at the active site of DvLysin (26) (Fig. 3A). As described for other NlpC/P60-containing peptidases (24–29), the catalytic C324 is located at the amino terminus of a helix packing against the central β-sheet that harbors H395 in its second strand and N415 in the third. In the PnpA structure, the thiol group of the catalytic cysteine is oxidized, resulting in the disruption of the characteristic C324 SD-H395 ND1 hydrogen bond and suggesting that the enzyme is in an inactive state (Fig. S4). As advanced for Bacteroides thetaiotamicron YkfC (BtYkfC) (26), oxidation of the catalytic cysteine most likely occurred during crystallization or exposure to X-rays (52), since recombinant PnpA from the same purification batch was used in biochemical assays and was catalytically active.

10.1128/mSphere.00736-20.6 FIG S4 Active site region of *Phdp* PnpA with superposed 2*Fo-Fc* electron density map (green mesh) contoured at 1.0 σ. The contact normally established between the imidazole ring of H395 and the side chain of N415 is represented by a dashed line, and the respective distance (in angstroms) is indicated. Download FIG S4, TIF file, 1.1 MB. Copyright © 2021 Lisboa et al. 2021 Lisboa et al. This content is distributed under the terms of the Creative Commons Attribution 4.0 International license.

In DvLysin, access to the catalytic cysteine occurs through a groove between the NlpC/P60 domain on one side and the c-clip helices αD and αE plus the SH3b1 domain on the other, with the RT loop from the SH3b1 domain closing one end of the groove (Fig. 3B) (26). While this topology is generally maintained in PnpA, the end of the groove opposite to the RT loop is also closed by strands β2 and β3 and the 3_10_ helix η4, creating a narrower access to the catalytic site (Fig. 3B). A minor difference is observed on the “wall” formed by the NlpC/P60 domain, wider in PnpA and closed by R414 and R452 in DvLysin (Fig. 3B). Besides the narrower entrance, two clusters of amino acids confer to the active site cavity of PnpA a more polar and hydrophobic nature than observed for DvLysin (Fig. 3C). However, extensive conservation of substrate-interacting residues between PnpA and DvLysin (Fig. 3C) suggests a similar interaction with *meso*-diaminopimelic acid (mDAP)-d-Ala from the stem peptide.

### PnpA is secreted by *Phdp* type II secretion system.

**FIG 4:**
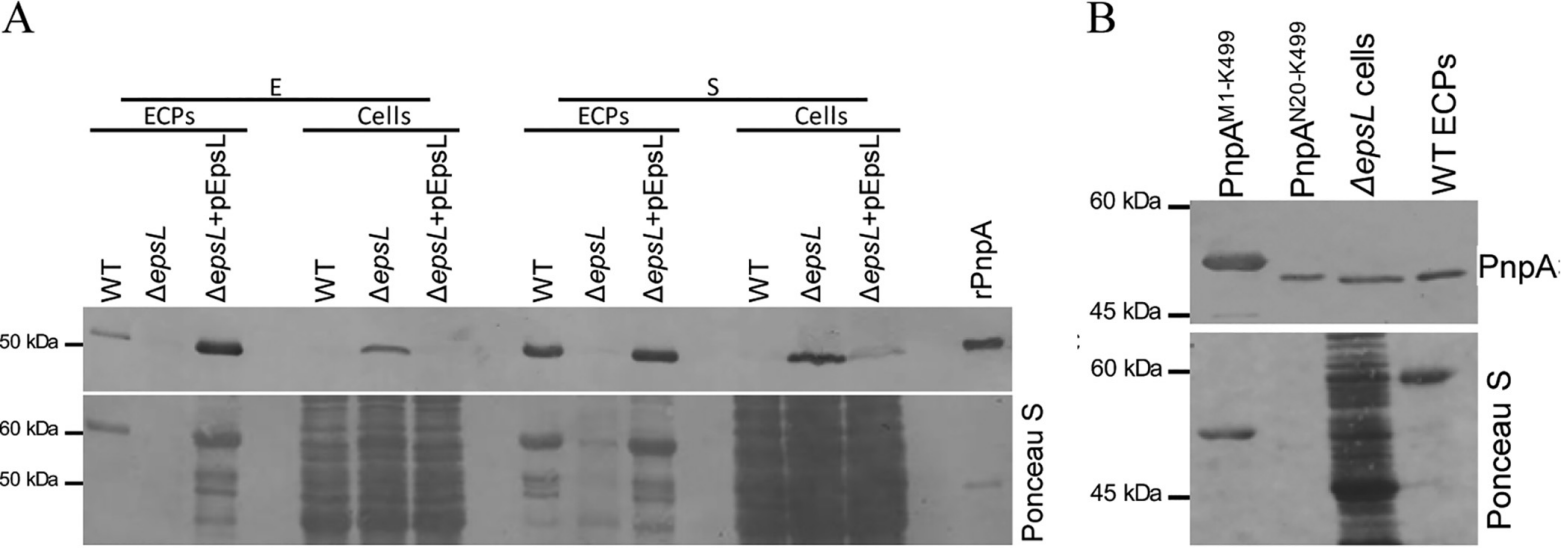
Secretion of PnpA is dependent on the type II secretion system (T2SS). (A) Wild type, *ΔepsL*, and Δ*epsL* complemented (Δ*epsL* + pEpsL) strains grown to an OD_600_ of 0.5 (exponential phase [E]) or 1.5 (stationary phase [S]). Extracellular products (ECPs) and bacterial pellets (Cells) were subjected to SDS-PAGE, and PnpA was detected by Western blotting (top panel). Recombinant PnpA (rPnpA; 0.2 μg) was used as a positive control. The bottom panel shows total protein loading (Ponceau S). The blot shown is representative of three independent experiments. (B) In *ΔepsL* cells, PnpA is retained at the periplasm. Western blotting of PnpA retained in *ΔepsL* cells and secreted by the WT bacteria (top panel). rPnpA containing and lacking the Sec signal peptide (PnpA^M1-K499^ and PnpA^N20-K499^, respectively) were run as references. Please note that PnpA retained in *ΔepsL* cells migrates similarly to the PnpA secreted by the WT bacteria, confirming the removal of the signal peptide and, thus, the periplasmic localization of PnpA in *ΔepsL* cells. The bottom panel shows total protein loading (Ponceau S).

PnpA possesses a typical Sec signal peptide and was identified in the culture supernatants of exponentially growing *Phdp* cultures, suggesting that it could be actively secreted by the bacteria. Many proteins that are transported via the Sec system into the periplasm are secreted across the outer membrane through a type II secretion system (T2SS) (53, 54). Recently, it was shown that *Phdp* contains a functional T2SS (44) and that deletion of *epsL*, which encodes an inner membrane-spanning protein that establishes a critical link between the cytoplasmic and periplasmic parts of that system (55), abolishes the secretion of AIP56 (44). To test the involvement of the T2SS of *Phdp* in PnpA secretion, the presence of PnpA in total cell lysates and extracellular products of wild-type (WT), Δ*epsL*, and Δ*epsL +* pEpsL *Phdp* was analyzed by Western blotting (Fig. 4). PnpA was detected in ECPs, but not in total cell lysates of the WT strain, confirming that it is a secreted protein (Fig. 4A). In contrast, in the Δ*epsL* strain, PnpA was retained in the cell, likely in the periplasm (Fig. 4B), confirming the involvement of T2SS in PnpA secretion.

### PnpA has specificity for the ***γ***-d-glutamyl-*meso*-diaminopimelic acid bond.

**FIG 5:**
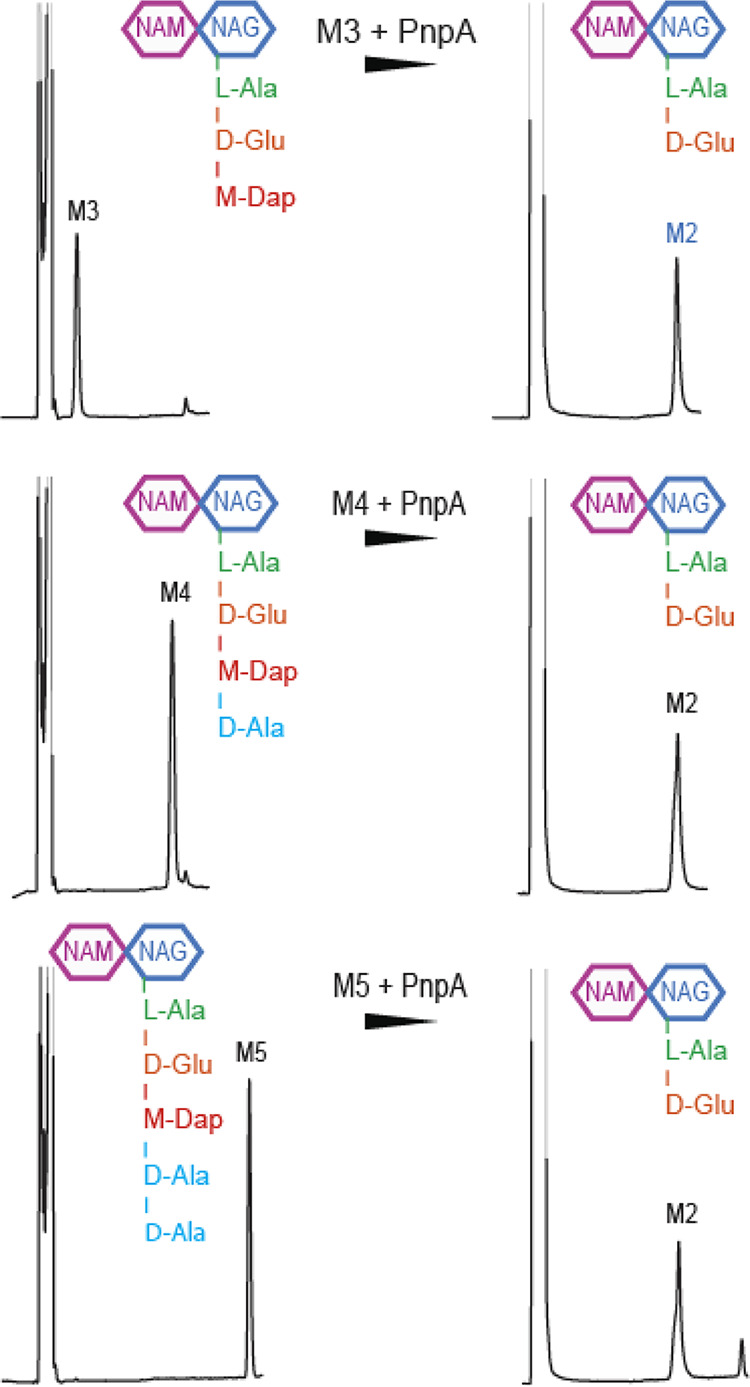
PnpA cleaves monomeric muropeptides M3, M4, and M5. HPLC profiles of each muropeptide at time zero (left) and after 3 h of incubation (right) with 50 μg ml^−1^ recombinant PnpA (rPnpA). Note that after incubation with PnpA, M2 was always obtained as a product. M3, GlcNAc-MurNAc-l-Ala-d-Glu-mDAP-d-Ala-d-Ala; M4, GlcNAc-MurNAc-l-Ala-d-Glu-mDAP-d-Ala; M5, GlcNAc-MurNAc-l-Ala-d-Glu-mDAP-d-Ala-d-Ala.

To investigate the PnpA enzymatic activity toward PG muropeptides and define its substrate specificity, recombinant PnpA was incubated with monomeric trimuropeptides (M3; GlcNAcMurNAc-l-Ala-d-Glu-mDap), tetramuropeptides (M4; GlcNAc-MurNAc-l-Ala-d-Glu-mDap-d-Ala), and pentamuropeptides (M5; GlcNAc-MurNAc-l-Ala-d-Glu-mDap-d-Ala-d-Ala) and the cleavage product(s) analyzed by high-performance liquid chromatography (HPLC) (Fig. 5). PnpA converted all tested muropeptides to dipeptides (M2; GlcNAc-MurNAc-l-Ala-d-Glu), suggesting it cleaves specifically γ-d-glutamyl-*meso*-diaminopimelic acid bond of monomeric muropeptides.

### PnpA does not hydrolyze *Phdp* peptidoglycan.

**FIG 6:**
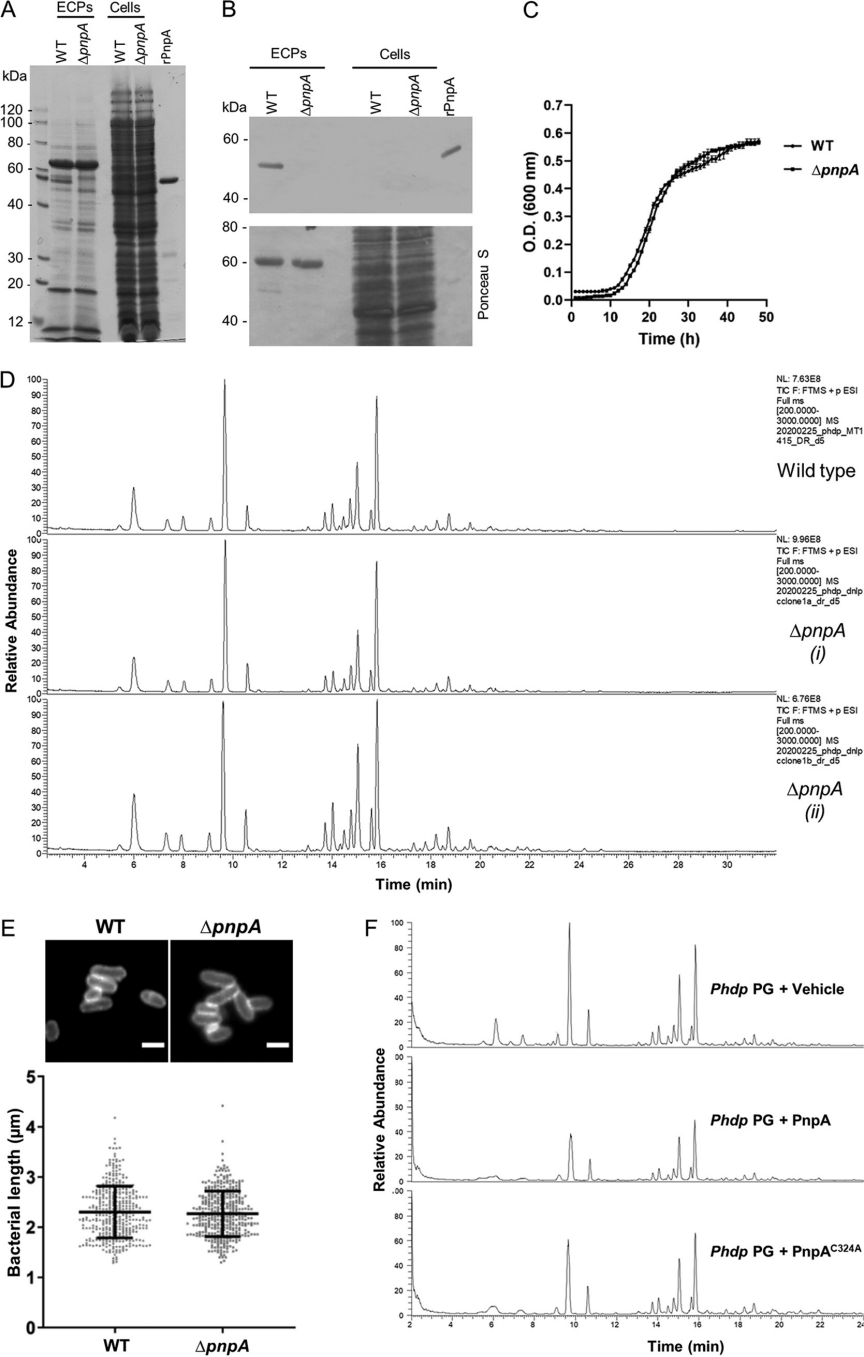
PnpA does not hydrolyze *Phdp* peptidoglycan. (A) SDS-PAGE of extracellular products (ECPs) and bacterial pellets (Cells) from WT and *ΔpnpA Phdp*. ECPs equivalent to 1.5 ml and cells equivalent to 0.3 ml of early stationary culture were separated by 12% SDS-PAGE and stained with Coomassie blue. Recombinant PnpA (rPnpA; 2 μg) was used as a reference. The gel shown is representative of two independent experiments. (B) Western blotting detection of PnpA in WT and *ΔpnpA* strains (top panel; ECPs and cells equivalent to 0.3 ml of culture). rPnpA (0.2 μg) was used as a control. The bottom panel shows total protein loading (Ponceau S). The blot shown is representative of three independent experiments. (C) Deletion of *pnpA* does not affect bacterial growth. *Phdp* MT1415 and MT1415*ΔpnpA* strains were grown in TSB-1 at 25°C. Growth curves were generated from three replicates for each strain. The results shown are representative of two independent experiments. (D) Total ion current (TIC) of digested and reduced PG from wild-type *Phdp* MT1415 (top) and MT1415*ΔpnpA* [middle and bottom; (i) and (ii) correspond to two independent cultures of *Phdp* MT1415*ΔpnpA*]. (E) Deletion of *pnpA* does not affect bacterial morphology. Bacteria labeled with wheat germ agglutinin (WGA)-Alexa Fluor 488 (top panel; bars, 2 μm). The lengths of at least 150 bacteria from two independent experiments were measured and graphed (bottom panel, mean length ± standard deviation [SD] [error bar]). Statistical significance was tested by Student’s *t* test, and no differences were observed. (F) Total ion current (TIC) of digested and reduced PG of *Phdp* previously incubated with vehicle, PnpA, or catalytically inactive PnpA^C324A^; the corresponding reduced supernatants are shown in Fig. S6 in the supplemental material.

In order to evaluate the involvement of PnpA in *Phdp* cell wall biogenesis, a *Phdp* Δ*pnpA* strain was generated, and the absence of PnpA expression in the mutant strain was confirmed by SDS-PAGE and Western blotting (Fig. 6A and B). Bacterial growth was not affected in the Δ*pnpA* strain (Fig. 6C). In addition, no differences were detected in the composition of the peptidoglycan from the WT and Δ*pnpA* strains (Fig. 6D; Table 1). In agreement with this, both WT and Δ*pnpA* strains showed similar morphology (Fig. 6E). Moreover, PnpA did not display *in vitro* enzymatic activity against *Phdp* whole sacculus, since no differences in the muropeptide composition were detected after incubating the PG with active PnpA or inactive PnpA (Fig. 6F and Fig. S5A; Table 2). Altogether, these results suggest that PnpA is not enzymatically active toward intact *Phdp* PG.

**TABLE 1:** Structure, molecular mass, and quantity of muropeptides from wild type and Δ*pnpA Phdp* MT1415 strains

Category and target name	ReTi (min)^*a*^	Formula	Molecular mass^*b*^				Quantity of muropeptides (%) from *Phdp* strain^*c*^		
			M (neutral mass)	[M+nH]^n+^Exp.	[M+nH]^n+^Th.	Error (ppm)	MT1415 (WT)	Δ*pnpA* *(1)*	Δ*pnpA|* *(2)*
Monomers									
GMDipeptide	9.11	C_27_H_46_N_4_O_17_	698.2858	699.2936	699.2931	0.74	4.71	5.13	5.16
GMTripeptide	6.00	C_34_H_58_N_6_O_20_	870.3706	436.1929	436.1926	0.69	13.42	12.53	14.92
GMTripeptide + Gly	7.35	C_36_H_61_N_7_O_21_	927.3921	464.7040	464.7035	1.08	3.23	3.47	3.99
GMTripeptide-K	8.00	C_40_H_70_N_8_O_21_	998.4656	500.2406	500.2401	1.10	4.00	3.46	3.63
GMTetrapeptide-R	8.67	C_43_H_75_N_11_O_22_	1,097.5163	549.7629	549.7617	2.18	0.01	0.01	0.01
GMTripeptide-Mipa-mDap	10.58	C_44_H_77_N_9_O_23_	1,099.5132	550.7645	550.7639	1.09	4.96	6.39	6.41
GMTetrapeptide	9.66	C_37_H_63_N_7_O_21_	941.4077	471.7115	471.7111	0.85	27.21	28.86	21.83
GanhMDipeptide	17.10	C_27_H_42_N_4_O_16_	678.2596	679.2683	679.2669	2.12	0.13	0.12	0.11
GanhMTripeptide	13.73	C_34_H_54_N_6_O_19_	850.3444	851.3517	851.3517	0.06	0.56	0.63	0.48
GanhMTetrapeptide	16.27	C_37_H_59_N_7_O_20_	921.38149	922.3905	922.3888	1.89	0.25	0.27	0.14
Dimers									
GMTripeptide-GMTripeptide	14.03	C_68_H_114_N_12_O_39_	1,722.7306	862.3735	862.3726	1.03	3.55	2.94	4.92
GMTripeptide-GMTetrapeptide	14.75	C_71_H_119_N_13_O_40_	1,793.7677	897.8941	897.8911	3.30	3.55	3.11	3.40
GMTripeptide-GMTetrapeptide	15.03	C_71_H_119_N_13_O_40_	1,793.7677	897.8923	897.8911	1.26	8.67	7.95	10.03
GMTripeptide-GMTripeptide + Gly	13.72	C_70_H_117_N_13_O_40_	1,779.7521	890.8842	890.8833	1.01	1.08	1.00	1.73
GMTetrapeptide-GMTetrapeptide	15.81	C_74_H_124_N_14_O_41_	1,864.8048	933.4105	933.4097	0.86	15.10	15.07	13.18
GMTetrapeptide-GMTripeptide + G	14.47	C_73_H_122_N_14_O_41_	1,850.7892	926.4035	926.4019	1.76	1.41	1.44	1.52
GMPentapeptide-GMTetrapeptide	16.34	C_77_H_129_N_15_O_42_	1,935.842	968.9301	968.9283	1.86	0.13	0.16	0.06
GMTetrapeptide-AmDapE	11.00	C_52_H_87_N_11_O_28_	1,313.5722	657.7948	657.7934	2.16	0.02	0.01	0.02
GMTetrapeptide-AmDapE	11.40	C_52_H_87_N_11_O_28_	1,313.5722	657.7948	657.7934	2.16	0.04	0.02	0.04
GMTetrapeptide-AmDapE	11.96	C_52_H_87_N_11_O_28_	1,313.5722	657.7948	657.7934	2.16	0.08	0.09	0.05
GMTetrapeptide-AmDap	10.48	C_47_H_80_N_10_O_25_	1,184.5296	593.2721	593.2733	−2.06	0.02	0.02	0.02
GMTetrapeptide-AmDap	11.04	C_47_H_80_N_10_O_25_	1,184.5296	593.2731	593.2733	−0.37	0.26	0.31	0.11
GMTetrapeptide-AmDapEA	12.25	C_55_H_92_N_12_O_29_	1,384.6093	693.3133	693.3119	1.96	0.13	0.07	0.10
GanhMTripeptide-GMTripeptide	18.70	C_68_H_110_N_12_O_38_	1,702.7044	852.3603	852.3595	0.96	1.46	1.23	1.85
G[anhM]Tetrapeptide-G[M]Tripeptide (A-A2pm)	19.38	C_71_H_115_N_13_O_39_	1,773.7415	887.8795	887.8780	1.69	0.57	0.54	0.54
GMTetrapeptide-G(anhM)Tripeptide	19.58	C_71_H_115_N_13_O_39_	1,773.7415	887.8795	887.8780	1.69	1.20	1.06	1.25
G(anhM)Tetrapeptide-GMTripeptide	19.75	C_71_H_115_N_13_O_39_	1,773.7415	887.8795	887.8780	1.69	0.58	0.51	0.60
GanhMTetrapeptide-GMTetrapeptide	20.41	C_74_H_120_N_14_O_40_	1,844.7786	923.3986	923.3966	2.18	1.03	1.02	0.88
GanhMTripeptide-GanhMTetrapeptide	23.52	C_71_H_111_N_13_O_38_	1,753.7153	877.8675	877.8649	2.96	0.01	0.01	0.01
GanhMTripeptide-GanhMTetrapeptide	23.81	C_71_H_111_N_13_O_38_	1,753.7153	877.8675	877.8649	2.96	0.03	0.03	0.03
GanhMTetrapeptide-anhMTetrapeptide	24.40	C_74_H_116_N_14_O_39_	1,824.7524	913.3866	913.3835	3.39	0.01	0.01	0.01
TetrasaccharideTetra-GMTetra	17.31	C_93_H_154_N_16_O_53_	2,342.9847	782.0025	782.0022	0.38	0.27	0.28	0.29
Trimers									
GMTripeptide-GMTetrapeptide- GMTetrapeptide	18.25	C_108_H_180_N_20_O_60_	2,717.1649	906.7307	906.7289	1.93	0.91	0.83	1.15
GMTetrapeptide-GMTetrapeptide -GMTetrapeptide	18.74	C_111_H_185_O_61_N_21_	2,788.202	930.4092	930.4079	1.40	1.39	1.42	1.51

Cross-linking (%)							21.2	19.9	22.1
Avg glycan chain length							28.4	29.7	28.9

a ReTi, retention time.

b M, molecular mass.

c Data from two independent cultures of *Phdp* MT1415Δ*pnpA* are shown by *(1)* and *(2)*.

**TABLE 2:** Structure, molecular mass, and quantity of muropeptides from *Phdp* and V. vulnificus PG incubated with vehicle, PnpA, or PnpA^C324A^

Category and target name^*a*^	ReTi (min)^*b*^	Formula	Molecular mass^*c*^				Quantity of muropeptides (%)					
							*Phdp*			Vibrio vulnificus		
			M (neutral mass)	[M+nH]^n+^ exp.	[M+nH]^n+^ Th.	Error (ppm)	Vehicle	PnpA	PnpA^C324A^	Vehicle	PnpA	PnpA^C324A^
Monomers												
GMDipeptide	9.12	C_27_H_46_N_4_O_17_	698.2858	699.2929	699.2931	−0.29	5.22	6.87	5.20	0.03	22.58	0.93
GMTripeptide	6.12	C_34_H_58_N_6_O_20_	870.3706	436.1925	436.1926	−0.23	10.99	10.05	10.65	0.82	0.81	1.24
GMTripeptide +Gly	7.41	C_36_H_61_N_7_O_21_	927.3921	464.7035	464.7033	0.43	3.69	3.42	3.59	0.97	0.57	0.95
GMTripeptide-K	8.10	C_40_H_70_N_8_O_21_	998.4656	500.2406	500.2401	1.10	0.50	0.47	0.49	0.33	0.19	0.32
GMTetrapeptide-R	8.75	C_43_H_75_N_11_O_22_	1,097.5163	549.7629	549.7617	2.18	0.01	0.01	0.01	5.77	3.48	5.70
GMTripeptide-Mipa-mDap	10.62	C_44_H_77_N_9_O_23_	1,099.5132	550.7645	550.7639	1.09	9.29	9.08	9.20	0.34	0.21	0.33
GMTetrapeptide	9.69	C_37_H_63_N_7_O_21_	941.4077	471.7109	471.7111	−0.42	27.25	27.26	27.58	44.64	30.82	42.12
GanhMDipeptide	17.10	C_27_H_42_N_4_O_16_	678.2596	679.2674	679.2669	0.79	0.14	1.24	0.20	0.01	11.50	0.56
GanhMTripeptide	13.74	C_34_H_54_N_6_O_19_	850.3444	851.3513	851.3517	-0.41	0.44	0.03	0.46	0.13	0.00	0.18
GanhMTetrapeptide	16.30	C_37_H_59_N_7_O_20_	921.38149	922.3893	922.3888	0.59	0.13	0.01	0.12	2.21	0.03	1.93
Dimers												
GMTripeptide-GMTripeptide	14.04	C_68_H_114_N_12_O_39_	1,722.7306	862.3725	862.3726	−0.09	3.56	3.64	3.61	0.00	0.00	0.00
GMTripeptide-GMTetrapeptide	14.79	C_71_H_119_N_13_O_40_	1,793.7677	897.8906	897.8911	−0.60	2.95	2.56	2.63	0.14	0.04	0.14
GMTripeptide-GMTetrapeptide (A free)	15.05	C_71_H_119_N_13_O_40_	1,793.7677	897.8907	897.8911	−0.49	10.46	10.94	10.82	0.17	0.10	0.18
GMTripeptide-GMTripeptide + Gly	13.74	C_70_H_117_N_13_O_40_	1,779.7521	890.8830	890.8833	−0.34	1.42	1.48	1.50	0.01	0.01	0.01
GMTetrapeptide-GMTetrapeptide	15.82	C_74_H_124_N_14_O_41_	1,864.8048	933.4094	933.4097	−0.32	14.98	14.05	14.67	17.79	5.27	18.03
GMTetrapeptide-GMTripeptide + G	14.49	C_73_H_122_N_14_O_41_	1,850.7892	926.4017	926.4019	−0.18	1.20	1.22	1.25	0.46	0.12	0.49
GMPentapeptide-GMTetrapeptide	16.35	C_77_H_129_N_15_O_42_	1,935.842	968.9292	968.9283	0.98	0.09	0.10	0.10	0.00	0.00	0.00
GMTetrapeptide-AmDapE	11.07	C_52_H_87_N_11_O_28_	1,313.5722	657.7940	657.7934	0.94	0.03	0.02	0.08	0.00	0.00	0.01
GMTetrapeptide-AmDapE	11.54	C_52_H_87_N_11_O_28_	1,313.5722	657.7941	657.7934	1.09	0.06	0.05	0.09	0.00	0.00	0.00
GMTetrapeptide-AmDapE	12.00	C_52_H_87_N_11_O_28_	1,313.5722	657.7942	657.7934	1.25	0.09	0.05	0.09	0.02	0.01	0.02
GMTetrapeptide-AmDap	10.48	C_47_H_80_N_10_O_25_	1,184.5296	593.2721	593.2733	−2.06	0.06	0.19	0.05	0.01	1.42	0.02
**GMTetrapeptide-AmDap**	11.04	C_47_H_80_N10O_25_	1,184.5296	593.2731	593.2733	−0.37	0.13	0.63	0.13	0.01	11.02	0.40
GMTetrapeptide-AmDapEA	12.26	C_55_H_92_N_12_O_29_	1,384.6093	693.3124	693.3119	0.68	0.15	0.11	0.12	0.30	0.10	0.27
GanhMTripeptide-GMTripeptide	18.67	C_68_H_110_N_12_O_38_	1,702.7044	852.3593	852.3595	−0.20	0.94	0.86	1.02	0.01	0.01	0.01
G[anhM]Tetrapeptide-G[M]Tripeptide (A-A2pm)	19.37	C_71_H_115_N_13_O_39_	1,773.7415	887.8789	887.8780	1.01	0.42	0.41	0.39	0.69	0.21	0.68
GMTetrapeptide-G(anhM)Tripeptide	19.60	C_71_H_115_N_13_O_39_	1,773.7415	887.8784	887.8780	0.45	0.96	1.02	0.96	0.18	0.11	0.18
G(anhM)Tetrapeptide-GMTripeptide	19.75	C_71_H_115_N_13_O_39_	1,773.7415	887.8793	887.8780	1.46	0.49	0.47	0.59	0.07	0.04	0.09
GanhMTetrapeptide-GMTetrapeptide	20.40	C_74_H_120_N_14_O_40_	1,844.7786	923.3973	923.3966	0.77	0.78	0.74	0.79	20.13	7.71	19.90
GanhMTripeptide-GanhMTetrapeptide	23.45	C_71_H_111_N_13_O_38_	1,753.7153	877.8660	877.8649	1.25	0.60	0.12	0.66	0.01	0.00	0.01
GanhMTripeptide-GanhMTetrapeptide	23.80	C_71_H_111_N_13_O_38_	1,753.7153	877.8661	877.8649	1.37	0.06	0.04	0.07	0.02	0.01	0.02
GanhMTetrapeptide-GanhMTetrapeptide	24.40	C_74_H_116_N_14_O_39_	1,824.7524	913.3838	913.3835	0.33	0.01	0.01	0.01	3.17	0.75	3.33
TetrasaccharideTetra-GMTetra	17.31	C_93_H_154_N_16_O_53_	2,342.9847	782.0025	782.0022	0.38	0.42	0.38	0.42	0.58	0.21	0.56
**TetrasaccharideTetra-AmDap**	13.57	C_66_H_110_N_12_O_37_	1,662.7095	832.3627	832.3620	0.84	0.00	0.00	0.00	0.00	0.28	0.01
**GanhMTetrapeptide-AmDap**	16.84	C_47_H_76_N_10_O_24_	1,164.5034	583.2595	583.2590	0.86	0.00	0.02	0.00	0.00	2.07	0.32
Trimers												
GMTripeptide-GMTetrapeptide- GMTetrapeptide	18.21	C_108_H_180_N_20_O_60_	2,717.1649	906.7293	906.7289	0.45	1.22	1.23	1.23	0.15	0.06	0.16
GMTetrapeptide-GMTetrapeptide- GMTetrapeptide	18.69	C_111_H_185_O_61_N_21_	2,788.202	930.4081	930.4079	0.21	1.23	1.24	1.25	0.81	0.25	0.88

Cross-linking (%)							21.1	20.5	21.2	22.1	7.4	22.2
Avg glycan chain length							35.1	32.2	33.2	6.9	6.3	6.8

a PnpA degradation products absent from native PG are shown in boldface type.

b ReTi, retention time.

c M, molecular mass.

10.1128/mSphere.00736-20.7 FIG S5 PnpA degrades the peptidoglycan (PG) of Vibrio vulnificus (B) and V. anguillarum (C), but not the PG of Photobacterium damselae subsp. *piscicida* (A) or of other Gram-negative (D to H) and Gram-positive (I and J) bacteria. Unless stated otherwise, incubations were performed after treating the PG with α-amilase. In the case of *Phdp* and V. vulnificus, digestions of PG not treated with α-amilase were also performed, and no differences were obtained. Download FIG S5, TIF file, 0.5 MB. Copyright © 2021 Lisboa et al. 2021 Lisboa et al. This content is distributed under the terms of the Creative Commons Attribution 4.0 International license.

10.1128/mSphere.00736-20.8 FIG S6 Reduced supernatants from peptidoglycan (PG) of *Phdp* and Vibrio vulnificus incubated with PnpA. (A) Ion extract of *m*/*z* 922.3923 in V. vulnificus supernatant; (B) ion extract of *m*/*z* 922.3923 shown in panel A and its MS/MS fragmentation spectrum. Download FIG S6, TIF file, 0.7 MB. Copyright © 2021 Lisboa et al. 2021 Lisboa et al. This content is distributed under the terms of the Creative Commons Attribution 4.0 International license.

### PnpA has hydrolytic activity toward Vibrio anguillarum and Vibrio vulnificus PG.

**FIG 7:**
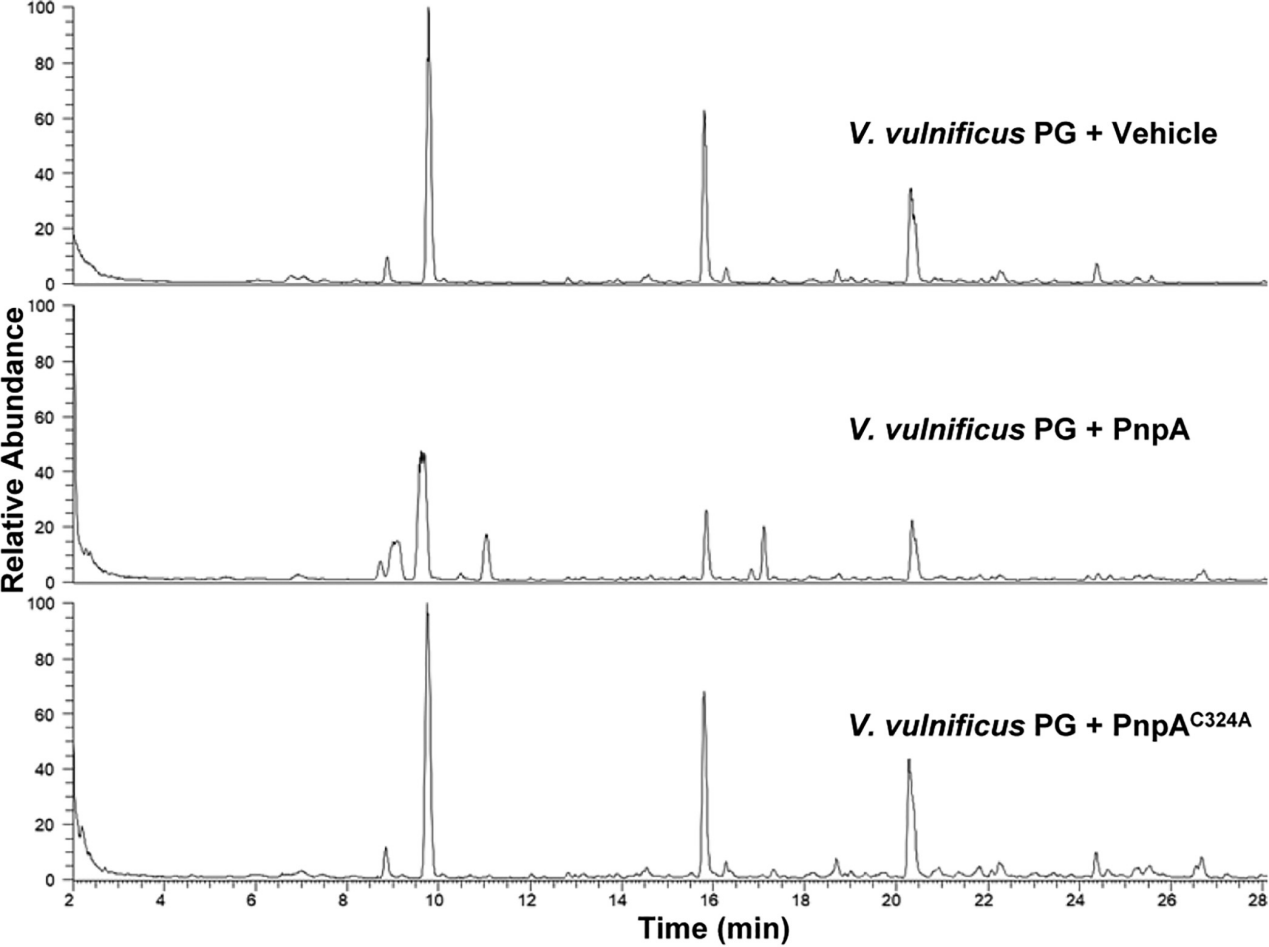
Total ion current (TIC) of digested and reduced PG of V. vulnificus previously incubated with vehicle, PnpA, or catalytically inactive PnpA^C324A^; the corresponding reduced supernatants are shown in Fig. S6.

The facts that PnpA is actively secreted into the extracellular medium and has no enzymatic activity for *Phdp* PG raised the possibility that it could cleave PG from other bacteria, functioning as a weapon against competing bacteria or as part of a mechanism to acquire nutrients, e.g., muropeptides from dead bacteria. To address this issue, whole sacculi from several Gram-positive or Gram-negative bacteria were isolated and incubated *in vitro* with recombinant PnpA or catalytically inactive PnpA (PnpA^C324A^) (Fig. 7 and Fig. S5B to J). Interestingly, only sacculi from V. anguillarum and V. vulnificus were sensitive to the action of PnpA (Fig. 7 and Fig. S5B and C and S6). Analysis of the insoluble sacculi resulting from digestion with PnpA showed the appearance of novel muropeptides, not present after incubation with inactive PnpA^C324A^ or vehicle (Fig. 7; Table 2). V. anguillarum and V. vulnificus PG present a very simple muropeptide composition with three major muropeptides, the monomer GM-tetrapeptide (GM4), the dimer GM4-GM4, and the anhydro-dimer (GM4-GanhM4 and GanhM4-GM4). The high proportion of anhydro-muropeptides indicates that V. vulnificus has a PG with short glycan chains (Table 2). PnpA treatment led to the appearance of four new muropeptides, GM2, GanhM2, GM4-mDapA, and GanhM4-mDapA. GM2 and GanhM2 products are consistent with the hydrolysis of the γ-d-glutamyl-*meso*-diaminopimelic acid bond. The presence of GM4-mDapA and GanhM4-mDapA are also consistent with the hydrolysis of a dimer or higher oligomers such as the major dimers GM4-GM4 and GM4-GanhM4 and the trimers GM3-GM4-GM4 and GM4-GM4-GM4 (Table 2) at the γ-d-glutamyl-*meso*-diaminopimelic acid bond at one of the 4-amino-acid stem peptides.

Analysis of the products released from the V. vulnificus PG identified two main tetrasaccharides substituted with the l-alanine-d-glutamate dipeptide (GM2-GanhM2) and/or a remain of the dimer cross-link (GM4-GanhM4-mDapA; Fig. 7 and Fig. S6; Table 3). Additionally, the GanhM2 monomer, the remains of the monomer stem peptide mDapA and of dimer cross-link mDapA-mDapA were also released, confirming that PnpA is indeed a γ-d-glutamyl-*meso*-diaminopimeate endopeptidase (Fig. 7 and Fig. S6; Table 3).

**TABLE 3:** Analysis of PG reduced supernatants from *Phdp* and Vibrio vulnificus after incubation with PnpA

Target name	ReTi (min)	Formula	Molecular mass				Supernatant^*a*^	
			M (neutral mass)	[M+nH]^n+^exp.	[M+nH]^n+^Th.	Error (ppm)	*Phdp* PG + PnpA	V. vulnificus + PnpA
mDap-Alanine+H_2_O	2.11	C_10_H_19_N_3_O_5_	261.1325	262.1391	262.1398	−2.67	4.20E + 06	6.95E + 07
mDap-Alanine-mDap- Alanine+H_2_O	2.27	C_20_H_36_N_6_O_9_	504.2544	505.2610	505.2617	−1.39	8.84E + 06	1.41E + 09
Nonreduced								
GanhMDipeptide	18	C_27_H_42_N_4_O_16_	678.2596	679.2674	679.2669	0.74	2.99E + 08	5.29E + 08
GanhMTetrapeptide	17.25	C_37_H_59_N_7_O_20_	921.38149	922.3893	922.3888	0.55	0.00E + 00	0.00E + 00
GM(Dipeptide)- GanhMTetrapeptide- mDapA-mDapA	20.36	C_84_H_135_N_17_O_44_	2,085.885	1,043.9495	1,043.9497	−0.19	0.00E + 00	2.31E + 08
GM(Dipeptide)- GanhMTetrapeptide-mDapA	21.64	C_74_H_118_N_14_O_40_	1,842.763	922.3888	922.3888	0.00	0.00E + 00	2.90E + 08
GM(Dipeptide)- GanhMDipeptide	22.84	C_54_H_84_N_8_O_32_	1,356.5192	679.2655	679.2669	−2.06	0.00E + 00	4.95E + 09
GM(Dipeptide)-GM(Dipeptide)- GanhMDipeptide	25.95	C_81_H_126_N_12_O_48_	2,034.7787	1,018.3970	1,018.3967	0.29	2.94E + 05	3.57E + 08

a Values indicate the intensity of the corresponding muropeptide by mass spectrometry analysis in arbitrary units.

In order to assess whether PnpA could inhibit the growth of competitor bacteria, the growth of V. vulnificus was monitored in the presence of PnpA (5 μg ml^−1^), and no growth inhibition was observed (Fig. S7A). To test the hypothesis that an additional factor secreted by *Phdp* could assist PnpA in reaching the PG, the growth of V. vulnificus was monitored in the presence of ECPs from wild-type or Δ*pnpA Phdp* (Fig. S7B) and in coculture experiments (Fig. S7C). No growth inhibition was observed in any of these experiments. Finally, it was tested whether PnpA was able to inhibit the growth of V. vulnificus in the presence of EDTA, an external membrane-permeabilizing agent used to mimic conditions that may be encountered in the host, and no effect on growth was observed (Fig. S7D).

10.1128/mSphere.00736-20.9 FIG S7 PnpA does not affect Vibrio vulnificus growth, and deletion of *pnpA* does not affect sea bass mortality after intraperitoneal (i.p.) infection. (A) Recombinant PnpA does not inhibit V. vulnificus growth. V. vulnificus strain CECT 4999 (Vv) was grown in TSA-1 for 24 h at 25°C and suspended in TSB-1 at an OD_600_ of 0.5 to 0.6. This suspension was inoculated in 1 ml TSB-1, TSB-1 plus 5 μg ml^−1^ PnpA or TSB-1 plus vehicle at 1:50 dilution in wells of a 24-well plate. The plate was incubated at 25°C with shaking, and the OD_600_ was read using a Synergy plate reader. Growth curves were generated from the three replicates for each condition. The results shown are representative of two independent experiments. (B) PnpA does not affect V. vulnificus growth in the presence of other extracellular products secreted by *Phdp*. *Phdp* MT1415 and MT1415*ΔpnpA* strains were grown in TSA-1 for 48 h, and V. vulnificus (Vv) (CECT 4999) was grown in the same medium for 24 h. MT1415 and MT1415*ΔpnpA* were suspended in TSB-1 at an OD_600_ of 0.5, inoculated 1:100 in TSB-1, and grown at 25°C until an OD_600_ of 0.8. ECPs were collected by centrifugation and passed through a 0.2-μm filter. Vv bacteria were suspended in TSB-1 at an OD_600_ of 0.5 and inoculated in 20 ml TSB-1 or in ECPs from MT1415 or MT1415*ΔpnpA* at 1:50 and grown at 25°C. The OD_600_ was measured at the indicated times and normalized for the OD_600_ measured at 1 h. The results shown are representative of two independent experiments. (C) Growth of V. vulnificus is not affected by coculture with *Phdp*. *Phdp* MT1415 and MT1415*ΔpnpA* strains were grown in TSA-1 plates for 48 h, and V. vulnificus (Vv) (CECT 4999) was grown in the same medium for 24 h. MT1415 WT and *ΔpnpA* were suspended in TSB-1 at an OD_600_ of 0.5, inoculated 1:50 in TSB-1, and grown overnight. The cultures were refreshed in TSB-1 to an OD_600_ of 0.25 and grown until an OD_600_ of 0.5. Vv was suspended in TSB-1 to an OD_600_ of 0.5, inoculated in the WT and *ΔpnpA* cultures at 1:50, and grown at 25°C for 6 h. Cocultures were serially diluted (1:10) in TSB-1 and spotted (10-μl dots) in TSA-1 or TCBS agar. Note that TCBS agar allows the growth of Vv but not of *Phdp*. The results shown are representative of two independent experiments. (D) PnpA does not affect V. vulnificus growth in the presence of the outer membrane permeabilizer EDTA. V. vulnificus grown in TSA-1 for 24 h at 25°C and suspended in TSB-1 at an OD_600_ of 0.5 to 0.6 was inoculated in 1 ml TSB-1, TSB-1 plus 0.10 mM EDTA, or TSB-1 plus 0.15 mM EDTA, or the same medium containing 30 μg ml^−1^ of PnpA or inactive PnpA^C324A^ at 1:50 dilution in an 24-well plate. TSB-1 (blank), TSB-1 plus 0.15 mM EDTA, and TSB-1 plus 30 μg ml^−1^ PnpA plus 30 μg ml^−1^ PnpA^C324A^ were used as controls. The plate was incubated at 25°C with shaking, and the OD_600_ was read using Synergy plate reader. Growth curves were generated from three replicates for the conditions containing Vv and from a single read for the controls. The results shown are from one experiment. (E) Deletion of *pnpA* does not affect sea bass mortality after i.p. infection. Cumulative mortality of juvenile sea bass (average weight of 19.63 ± 3.63 g and 20.35 ± 3.86 g for experiments 1 and 2, respectively) injected i.p with the indicated doses of wild-type or *ΔpnpA* strains. The study was carried out in accordance with European and Portuguese legislation for the use of animals for scientific purposes (Directive 2010/63/EU; Decreto-Lei 113/2013). The work was approved by Direcção-Geral de Alimentação e Veterinária (DGAV), the Portuguese authority for animal protection (reference 0421/000/000/2013). Fish were maintained in recirculating aerated sea water at 22°C and previously acclimatized to 23 to 24°C prior to infection. To prepare the inocula, bacteria grown in TSA-1 plates for 48 h at 25°C were suspended in TSB-1 at an OD_600_ of 0.4, and these suspensions were used to inoculate 100 ml TSB-1 at 1:100 dilution. The cultures were grown for 14 to 15 h at 25°C with shaking (160 rpm) until an OD_600_ of 0.5 to 0.6 was reached, centrifuged (3,220 × *g*, 15 min, 4°C), and resuspended in TSB-1 at an OD_600_ of 0.6. Groups of 10 juvenile sea bass were anesthetized with 0.06% (vol/vol) ethylene glycol monophenyl ether and i.p. injected with 100 μl of these suspensions. In experiment 2, 1:10 dilutions of the initial suspensions were also injected. CFUs of each inoculum were determined by plating serial dilution onto TSA-1. Download FIG S7, TIF file, 0.5 MB. Copyright © 2021 Lisboa et al. 2021 Lisboa et al. This content is distributed under the terms of the Creative Commons Attribution 4.0 International license.

## DISCUSSION

In this work, the structural and functional characterization of PnpA, an NlpC/P60 family peptidase secreted by Photobacterium damselae subsp. *piscicida* (*Phdp*) is reported. PnpA is not essential for *Phdp* cell wall biogenesis and does not cleave *Phdp* PG, but it degrades the PG of V. anguillarum and V. vulnificus, two bacterial species that share the same hosts and/or environment as *Phdp*. On the basis of these observations, it is proposed that PnpA may allow *Phdp* to fight competitors or to acquire nutrients from dead coinhabitants.

Many cysteine peptidases containing the NlpC/P60 domain were characterized thus far (1, 2, 24, 26, 30, 33–35), several of which display a four-domain organization similar to PnpA. However, until now, only the three-dimensional structure of DvLysin from Desulfovibrio vulgaris was reported, with a N-terminal “c-clip” or “N_NLPC_P60” stabilizing domain, two SH3b domains, and a C-terminal NlpC/P60 cysteine peptidase domain (26). Furthermore, among the known DvLysin and PnpA orthologs, only EcgA from Salmonella enterica serovar Typhimurium was functionally characterized (56). Although the three molecules are very similar (25 to 27% amino acid sequence identity) (see Fig. S8A in the supplemental material), DvLysin does not have the insertion found in PnpA and EcgA and that in PnpA closes the side of the catalytic groove opposed to the RT loop (Fig. 2 and 3 and Fig. S8B). Despite these differences, residues involved in substrate binding in DvLysin (26) are conserved in PnpA and EcgA (Fig. 3C and Fig. S8B), in agreement with their specificity for the γ-d-glutamyl-*meso*-diaminopimelic acid bond (Fig. 5) (26, 56). However, unlike DvLysin (26) and EcgA (56), which were more active toward tetra- and trimuropeptides, respectively, PnpA showed activity toward penta-, tetra-, and tripeptides (Fig. 5).

10.1128/mSphere.00736-20.10 FIG S8 Amino acid similarity and identity determined with EMBOSS (A) and amino acid sequence alignment (B) of PnpA, EcgA from Salmonella enterica serovar Typhimurium, and DvLysin from Desulfovibrio vulgaris Hildenborough. Alignment was performed using Clustal Omega (F. Madeira, Y. M. Park, J. Lee, N. Buso, T. Gur, N. Madhusoodanan, P. Basutkar, A. R. N. Tivey, S. C. Potter, R. D. Finn, R. Lopez, Nucleic Acids Res 47:W636–W641, 2019, https://doi.org/10.1093/nar/gkz268). Strictly conserved residues are in white on a red background. Partially conserved amino acids are boxed. Secondary structure elements from PnpA are indicated above the alignment. The numbers (green) below the sequences indicate the disulfide bridge present in PnpA. The cyan box highlights the antiparallel β-sheet (β2 and β3) and a 3_10_ helix (η4) in the c-clip domain of PnpA, EcgA that is absent in DvLysin. This figure was generated using the ESPript server (ESPript at http://espript.ibcp.fr; X. Robert, P. Gouet, Nucleic Acids Res 42:W320−W324, 2014, https://doi.org/10.1093/nar/gku316). Download FIG S8, TIF file, 2.5 MB. Copyright © 2021 Lisboa et al. 2021 Lisboa et al. This content is distributed under the terms of the Creative Commons Attribution 4.0 International license.

So far, the cellular localization of DvLysin and its function in *D. vulgaris* cell wall biogenesis remain unknown (26). Regarding EcgA, its expression is induced when *S.* Typhimurium is inside eukaryotic cells, localizing in the inner and outer membranes where it plays a role in PG remodeling and contributes to *S.* Typhimurium virulence (56). In contrast, PnpA is secreted by the T2SS into the extracellular medium (Fig. 4), and deletion of *pnpA* does not affect *Phdp* growth, PG composition, and morphology (Fig. 6C to E). Accordingly, PnpA has no *in vitro* hydrolytic activity toward *Phdp* sacculi (Fig. 6F and Fig. S5A). Altogether, these results suggest that PnpA is not involved in *Phdp* cell wall biogenesis.

**FIG 8:**
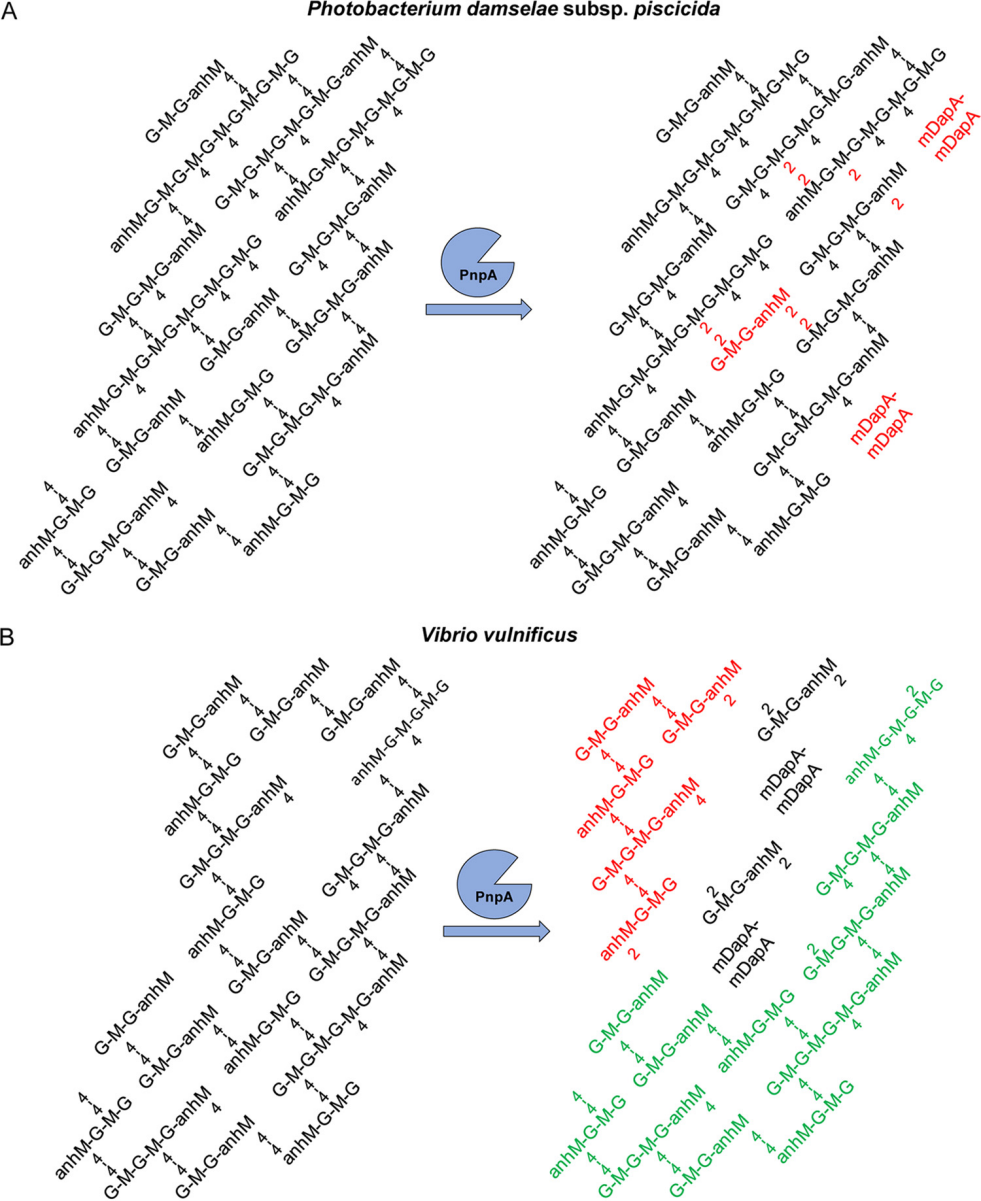
Structural model of *Phdp* (A) and V. vulnificus (B) PG suggesting that the higher degree of cross-linking of very short glycans in the V. vulnificus PG may explain its vulnerability to the enzymatic activity of PnpA.

The resistance of *Phdp* PG to the activity of PnpA is in sharp contrast with the ability of PnpA to hydrolyze penta-, tetra-, and trimuropeptides, since the chemical composition of *Phdp* PG suggested that it would be a target of PnpA. This unexpected resistance to PnpA was not exclusively observed with PG from *Phdp*, as it also occurred when using sacculi from multiple bacterial species (Fig. S5). In fact, PGs from V. anguillarum and V. vulnificus were sensitive to the activity of PnpA, despite having a PG composition characteristic of Gram-negative bacteria and similar to the composition of some PG shown to be resistant to PnpA hydrolysis. Hence, PnpA specificity for V. anguillarum and V. vulnificus PG cannot be explained by their muropeptide composition and may be related to specific three-dimensional features of the PG mesh. Accordingly, the analysis of the V. anguillarum and V. vulnificus PG composition shows that these two species have a high proportion of anhydro-muropeptides, a trademark of the end of glycans, indicating that their glycan chains are rather short compared to other Gram-negative bacteria. Consequently, structural analysis of the products released upon incubation of the sacculi of V. anguillarum and V. vulnificus with PnpA identified a high proportion of the tetrasaccharide GM2-GanhM2. This suggests that the PG of V. anguillarum and V. vulnificus is enriched in tetrasaccharides. The simultaneous release of mDapA-mDapA suggests that these tetrasaccharides are linked to the rest of the PG by one or even two cross-links. These results combined with the rather simple muropeptide composition of V. anguillarum and V. vulnificus suggest that the vulnerability of V. anguillarum and V. vulnificus to PnpA might arise from the fact that their PGs rely on very short, highly cross-linked glycans. Hence, hydrolysis of the stem peptides by PnpA leads to a rapid destruction of the PG layer while in other Gram-negative species, because they have much longer glycans, PG integrity can be maintained by multiple dimers along the same glycan chain (Fig. 8).

Expression levels of *pnpA* in standard culture conditions do not vary between the logarithmic and stationary growth phases (Fig. S1) but increase under iron-limited conditions or in response to oxidative stress (57). However, *in vivo*, no changes in *pnpA* expression were detected after intraperitoneal infection of sole (Solea senegalensis) with *Phdp* (57), and deletion of *pnpA* did not affect *Phdp* virulence in a sea bass (Dicentrarchus labrax) intraperitoneal infection model (Fig. S7E). This suggests that PnpA is likely dispensable at late systemic phases of *Phdp* infection but does not exclude a role of PnpA in earlier stages of the infection. It is known that, during the systemic phase of *Phdp*-induced disease, the exotoxin AIP56 plays a major role by neutralizing host phagocytic defenses (43–45, 47, 58). However, little is known about the early stages of the infection. Here, it is shown that PnpA specifically hydrolyzes the sacculi of V. anguillarum and V. vulnificus (Fig. 7, Fig. S5B and C, and Fig. S6), two other enterobacteria present in the marine environment (59–61) and, at least in the case of V. anguillarum, reported as infecting the same hosts as *Phdp* (37, 61). This suggests that before reaching the systemic phase, *Phdp* may secrete PnpA to gain competitive growth advantage over bacteria sharing a complex community environment, such as the gastrointestinal tract, or to obtain nutrients in an environment where nutrient scarcity can compromise its survival, either inside the host or in water or sediment (40). These strategies have been first described for Gram-positive bacteria (21, 23), which have their PG exposed on the cell surface, accessible to secreted PG hydrolases (10, 11, 22). Gram-negative bacteria, despite having their PG protected by the outer membrane, can inject PG hydrolases, including NlpC/P60 family peptidases, into the periplasm of neighboring bacteria through type VI secretion systems (10, 17, 19, 20). The examples of using bacterial exohydrolases to target Gram-negative competitors are restricted to predatory bacteria such as myxobacteria (11) and Bdellovibrio bacteriovorus (18). Another example where PG hydrolases are secreted to eliminate competing bacteria is that reported for the urogenital pathogenic protozoan Trichomonas vaginalis (16), which has acquired by lateral genetic transfer two genes of bacterial origin encoding NlpC/P60 endopeptidases that the parasite secretes to degrade bacterial PG and thus outcompete bacteria from mixed cultures (16). However, it remains unclear how these exohydrolases reach the PG of the Gram-negative targets. Here, it was also not clarified how PnpA reaches the PG in V. vulnificus and V. anguillarum cell wall, since no growth inhibition was detected in several *in vitro* tests with V. vulnificus (Fig. S7), suggesting that the access of PnpA to the periplasm of competing bacteria may depend on conditions present at specific stages of the *Phdp* life cycle, when *Phdp* and competitors meet.

## MATERIALS AND METHODS

### Bacterial strains and culture conditions.

Photobacterium damselae subsp. *piscicida* (*Phdp*) virulent strain MT1415 isolated from sea bass in Italy (45) was cultured at 25°C in tryptic soy broth (TSB) or tryptic soy agar (TSA) supplemented with NaCl to a final concentration of 1% (wt/vol) (TSB-1 and TSA-1, respectively). The Δ*epsL* and Δ*pnpA* strains were cultured under the same conditions as the wild type. Δ*epsL* + pEpsL and Δ*pnpA* + pPnpA complemented strains were cultured in TSB-1 or TSA-1 supplemented with 10 μg ml^−1^ of gentamicin (TSB-1_Gm_ and TSA-1_Gm_, respectively). Stocks of bacteria were maintained at –80°C in TSB-1 supplemented with 15% (vol/vol) glycerol. To obtain growth curves, bacteria grown on agar plates for 48 h were suspended in TSB-1 or TSB-1_Gm_ at an optical density at 600 nm (OD_600_) of 0.5 to 0.6. These suspensions were inoculated in 20 ml TSB-1 (1:100 dilution). One-milliliter aliquots were removed (in triplicate) and transferred to 24-well culture plate, and the OD_600_ was determined kinetically (1 point/h) using a BioTek Synergy 2 spectrofluorometer (BioTeK U.S., Winooski, VT, USA) at 25°C with continuous slow agitation, for 60 to 70 h. Growth curves were constructed using GraphPad Prism software (La Jolla, CA, USA).

### Construction of Δ*pnpA* strain.

An in-frame (nonpolar) deletion of the almost complete *pnpA* coding sequence was constructed following an allelic exchange procedure as previously described (62). In brief, the 3′ and 5′ flanking sequences were PCR amplified using suitable primers (Mut_NlpC_1Eco [5′-GCGAATTCGTTTCGATGCGCTGATTAAT-3′], Mut_NlpC_2Bam [5′-GCGGATCCAGCAAAACATCAACAAGTCA-3′], Mut_NlpC_3Bam [5′-GCGGATCCATAGTTGGTTAATAATGCTA-3′], and Mut_NlpC_4Xba [5′-GCTCTAGATCAC-GATGGAATAGATAACT-3′] [restriction sites are underlined]). The PCR products were ligated to obtain an in-frame deletion of ca. 90% of the PnpA coding sequence. The deleted allele construction was cloned into the suicide vector pNidKan containing the *sacB* gene, which confers sucrose sensitivity, and R6K *ori*, which requires the *pir* gene product for replication. The plasmid containing the deleted allele was transferred from Escherichia coli S17-1-λ*pir* into the rifampin-resistant derivative of *Phdp* MT1415 by drop mating for 24 h on TSA plates prepared with seawater. Cells were then scrapped off the plate and selected on TSA supplemented with kanamycin (Kan) (50 μg ml^−1^) for plasmid integration. A selected Kan^r^ clone was further selected for sucrose resistance (15% [wt/vol]) for a second recombination event. This led to *Phdp* Δ*pnpA* mutant strain, which was tested by PCR to verify the correct allelic exchange.

### Bacterial cell extracts and extracellular products.

*Phdp* was grown in TSB-1 at 25°C with shaking (160 rpm) and centrifuged (6,000 × *g*, 5 min, 4°C), and the pellets (total cell extracts) and culture supernatants were collected. Supernatants were filtered (0.22 μm) to obtain extracellular products (ECPs). For SDS-PAGE, proteins in the ECPs were precipitated with trichloroacetic acid (TCA) as previously described (45).

### PnpA identification.

ECPs from *Phdp* strain MT1415 were subjected to SDS-PAGE followed by Coomassie blue staining. A protein band of approximately 55 kDa was analyzed by MALDI-TOF MS in a 4800 Proteomics Analyzer (Applied Biosystems) at TOPLAB GmbH. The MS data were used for a Mascot search against the NCBInr sequence database.

### Draft genome sequence of *Phdp* MT1415 and genomic context of *pnpA* locus.

To delete the PnpA-encoding gene in *Phdp* MT1415, it was necessary to obtain at least 2 kb of upstream and downstream sequences free of repetitive insertion sequence elements that would compromise the specific recombination steps during allelic exchange. Therefore, the draft genome sequence of strain MT1415 was obtained, using an Illumina platform as previously described (48) and deposited in the GenBank database under accession number SUMH00000000. A comparative analysis was conducted by retrieving the genomic contexts of *pnpA* genes in different *Phdp* and Photobacterium damselae subsp. damselae (*Phdd*) isolates whose draft or complete genomes are available in the GenBank database. The GenBank locus tag numbers of the *pnpA* homologues used in this analysis are VDA_000779 (*Phdd* type strain CIP 102761), PDPUS_2_00834 (*Phdp* 91–197), PDPJ_2_00460 (*Phdp* OT-51443), BEI67_17705 (*Phdp* L091106-03H), and BDMQ01000002 (*Phdp* DI21). For the *pnpA* negative *Phdd* strain RM-71, the draft genome sequence as a source of homologous flanking DNA sequences was used (accession number NZ_LYBT00000000.1). The DNA sequences were handled with Vector NTI 10.3.0 sequence editor (Invitrogen).

### Recombinant PnpA.

The *pnpA* open reading frame (ORF) (GenBank accession number TJZ86030.1) was amplified from *Phdp* MT1415 genomic DNA using *Pfu* DNA polymerase (Thermo Scientific) and primers 5′-cgcccATGGATATAAATAAACATTTAATGC-3′ and 5′-gcgctcgagTTTTTCAAATAGATATTTTTC-3′ (target sequences are in uppercase letters) and cloned into pET28a(+) using the NcoI and XhoI restriction sites, in frame with a C-terminal 6×His tag. Mutation of C^324^ to alanine was achieved by site-directed mutagenesis by inverse PCR using Q5 high fidelity DNA polymerase (New England BioLabs), pET28-PnpA as the template, and primers (5′-GCCTCTGGTTTATTAAAAAGGTTATTCAGC-3′ and 5′-ATCATTATTGAAATC-CATTCCCCC-3′). Proteins were expressed in E. coli BL21(DE3) CodonPlus-RIL (Stratagene). Four liters of LB medium with 50 μg ml^−1^ kanamycin and 25 μg ml^−1^ chloramphenicol were inoculated with pET28-PnpA- or pET28-PnpA^C324A^-transformed bacteria and incubated at 37°C until an OD_600_ of 0.6 to 0.8 was reached. Cultures were cooled at 17°C for 30 min, followed by the addition of 0.5 mM isopropyl-β-d-thiogalactopyranoside (IPTG) to induce protein expression. After 20 h, cells were harvested by centrifugation, resuspended in 50 mM Bis-Tris (pH 6.5) and 500 mM NaCl, and sonicated. Lysates were centrifuged (34,957 × *g*, 30 min, 4°C), and the soluble fraction was applied to a nickel-nitrilotriacetic acid (Ni-NTA) column (ABT), followed by anion-exchange chromatography (Bio-Scale Mini Macro-Prep High Q; Bio-Rad). Fractions containing the recombinant proteins were pooled and injected into a size exclusion chromatography column (Superose12 10/300 GL; GE Healthcare) equilibrated with 50 mM Bis-Tris (pH 6.5) and 500 mM NaCl. Fractions containing the desired protein were pooled, concentrated to 6 to 7 mg ml^−1^, frozen in liquid nitrogen, and stored at –80°C. Protein concentration was determined in a NanoDrop ND-1000 UV-visible (UV-Vis) spectrophotometer (Thermo Fisher Scientific) considering the extinction coefficient and the molecular weight calculated with the ProtParam tool (https://web.expasy.org/protparam/).

### Reverse transcription and quantitative PCR (qRT-PCR).

Total RNA was isolated from exponential (OD_600_ of 0.4) and stationary (OD_600_ of 1.2) cultures of *Phdp* strain MT1415. Bacterial pellets were resuspended in 25 mM Tris buffer supplemented with 20% (wt/vol) glucose and 0.5 M EDTA (pH 8.0) and lysed with phenol acid and glass beads by vortexing (4°C, 20 min). Lysates were centrifuged at 16,000 × *g* (4°C, 5 min), and the top liquid phase was collected. RNA was extracted using the TripleXtractor reagent (Grisp) and treated with DNase I (Turbo DNA-free; Ambion) following the manufacturer’s recommendations. RNA purity and integrity were verified by 1% (wt/vol) agarose gel electrophoresis in an Experion automated electrophoresis system (Bio-Rad). One microgram of RNA was reverse transcribed into cDNA (iScript kit; Bio-Rad). Quantitative real-time PCR was performed in 20-μl reaction mixtures containing 1 μl cDNA, 10 μl *iTaq* Universal SYBR green Supermix (Bio-Rad Laboratories), and 0.25 μM primers (PnpA forward primer [5′-GGATTTGGCTACCTCGTTCA-3′], PnpA reverse primer [5′-CCCACGGAG-CATTAAACATT-3′], 16S forward primer [5′-AACTGGCAGGCTAGAGTCTT-3′], and 16S reverse primer [5′-CACAACCTCCAAGTAGACAT-3′]), using the following protocol: 1 cycle at 95°C (3 min) and 40 cycles with 1 cycle consisting of 95°C (20 s), 51°C (15 s), and 72°C (30 s). For each condition, three biological replicates were analyzed, each of which had three technical replicates. Data were normalized to the expression values of the housekeeping gene (16S rRNA) and analyzed by the comparative threshold (ΔΔ*C_T_*) method.

### Anti-PnpA antibody.

The quail anti-PnpA antibody was produced at HenBiotech (catalog no. H003; HenBiotech, Coimbra, Portugal) using recombinant PnpA as the immunizing antigen. Quail IgYs were purified from an egg-yolk pool (IgY grade II/polyethylene glycol [PEG]).

### SDS-PAGE and Western blotting.

Bacterial cell pellets and ECPs were solubilized in loading buffer (50 mM Tris-HCl [pH 8.8], 2% [wt/vol] SDS, 0.05% [wt/vol] bromophenol blue, 10% [vol/vol] glycerol, 2 mM EDTA, and 100 mM dithiothreitol [DTT]) and subjected to SDS-PAGE (63). Proteins were stained with Coomassie blue or transferred onto nitrocellulose membranes. Transfer efficiency and protein loading were controlled by Ponceau S staining. Membranes were blocked with 5% (wt/vol) skim milk in Tris-buffered saline (TBS) containing 0.1% (vol/vol) Tween 20 (TBS-T), incubated with the anti-PnpA quail antibody (1:10,000 dilution) in blocking buffer followed by incubation with an anti-chicken alkaline phosphatase-conjugated secondary antibody (catalog no. A9171; Sigma) (1:10,000 dilution) and nitroblue tetrazolium (NBT)/5-bromo-4-chloro-3-indolylphosphate (BCIP) development.

### Crystallization.

Initial crystallization hits for PnpA were identified by high-throughput screening performed at the HTX Lab of the EMBL Grenoble Outstation (Grenoble, France). Crystallization experiments for refinement of the initial conditions were carried out using the hanging drop vapor diffusion method at 20°C. Crystals were obtained by mixing protein solution (6.7 mg ml^−1^ in 50 mM Bis-Tris [pH 6.5] and 500 mM NaCl) with an equal volume of crystallization solution (100 mM imidazole [pH 8.0], 15% [wt/vol] polyethylene 8000 [PEG 8K]). Crystals appeared after 24 to 48 h. The crystals were cryo-protected by sequential transfer into their crystallization condition with increasing concentrations of ethylene glycol (up to 30% [vol/vol]) and then flash-frozen in liquid nitrogen prior to data collection.

### Data collection, structure solution, and refinement.

Diffraction data were collected at beamline Proxima-1 of Synchrotron SOLEIL (Saint-Aubin, France) (64) on a Dectris Pilatus 6M detector (750 images, 0.2° rotation, 0.2-s exposure) and indexed and integrated with XDS (65). Space group determination, data scaling, and merging were performed with POINTLESS and AIMLESS from the CCP4 program suite (66). The structure of PnpA was solved by molecular replacement with Phaser MR as implemented in the CCP4 program suite (66, 67) using the coordinates of a putative gamma-d-glutamyl-l-diamino acid endopeptidase from Desulfovibrio vulgaris Hildenborough (DvLysin, PDB entry 3M1U, 26% sequence identity) as the search model. Phase refinement and initial model building were performed using ARP/wARP (68). Model completion and refinement were done iteratively with COOT (69) and Phenix.refine (70, 71), respectively. Refinement and structure validation statistics are summarized in Table S1 in the supplemental material. All illustrations of macromolecular models were produced with PyMOL (72). The experimental data were deposited with the Structural Biology Data Grid (73) under accession number https://doi.org/10.15785/SBGRID/736.

### *In vitro* muropeptide cleavage assays.

To investigate the PnpA enzymatic activity toward PG muropeptides, isolated M3 (GlcNAc‐MurNAc‐l‐Ala‐d‐Glu‐mDAP), M4 (GlcNAc‐MurNAc‐l‐Ala‐d‐Glu‐mDAP‐d‐Ala), and M5 (GlcNAc‐MurNAc‐l‐Ala‐d‐Glu‐mDAP‐d‐Ala‐d‐Ala) muropeptides from Salmonella enterica were incubated with 50 μg of PnpA in 50 mM Tris (pH 8.0) and 300 mM NaCl for 3 h at 37°C. The products of the reaction were analyzed by reverse-phase HPLC (Waters 1525 system) as previously described (56).

### Peptidoglycan (PG) purification.

Bacteria were grown in TSB-1 at 25°C with shaking (160 rpm) to exponential (OD_600_ of 0.4 to 0.5) or stationary (OD_600_ of 1.2 to 1.4) phases. Bacterial cells (∼10^11^) were centrifuged (4,200 × *g*, 10 min, room temperature [rt]), washed twice and resuspended in phosphate-buffered saline (PBS), and immediately mixed 1:1 (vol/vol) with a boiling solution of 8% SDS, drop by drop. Boiling was maintained for 8 h with stirring, followed by overnight incubation at rt. Samples were centrifuged (150,000 × *g*, 40 min, 4°C), the pellets were washed three times with ultrapure water (150,000 × *g*, 40 min, 4°C), resuspended in 10 mM Tris (pH 7.6) and 0.06% (wt/vol) NaCl with or without 100 μg ml^−1^ α-amylase, and incubated at 37°C for 90 min. Samples were treated for 2 h at 60°C with 100 μg ml^−1^ pronase E preactivated by incubation in the same buffer for 60 min at 60°C. Pronase E digestion was stopped by adding SDS (5.3% [wt/vol] final concentration) and heating at 100°C for 20 min. PG was recovered by centrifugation (300,000 × *g*, 10 min) and washed with ultrapure water.

### Analysis of *Phdp* PG composition and PG cleavage assays.

To analyze the PG composition of the *Phdp* MT1415 and MT1415Δ*pnpA* strains, PGs were purified as described above, digested overnight at 37°C in sodium phosphate buffer supplemented with 100 IU of mutanolysin from Streptomyces globisporus (ATCC 21553; Sigma), and reduced with NaH_4_B. After 30 min at rt and centrifugation, the reduced muropeptides were diluted in acidified water with formic acid (FA) and analyzed by high-performance liquid chromatography (HPLC) or HPLC/high-resolution mass spectrometry (HRMS). HPLC/HRMS was performed on an Ultimate 3000 UHLPC system coupled to a quadrupole orbitrap mass spectrometer (qExactive Focus; Thermo Fisher Scientific). Reduced muropeptides were eluted on an C_18_ analytical column (Hypersil gold aQ; 1.9 μm, 2.1 × 150 mm) held at 50°C under a 200 μl min^−1^ flow rate. A binary solvent system composed of acidified water (H_2_O + 0.1% FA; mobile phase A) and acidified acetonitrile (CH_3_CN + 0.1% FA, mobile phase B) was used for chromatographic separation. The composition was linearly increased to 12.5% mobile phase B over 25 min, increased to 20% mobile phase B for 5 min, and held for an additional 5 min. It was then stepped down to 0% over and held for 10 min to return initial conditions.

Exactive Focus was operated under electrospray ionization in positive mode and data-dependent acquisition mode (ddMS2) control by Xcalibur 4.0. For structural confirmation of muropeptides, higher-energy collisional dissociation (HCD) fragmentation was set up with a normalized collision energy at 20%. Data were processed both with the software TraceFinder 3.3 (Thermo Fisher Scientific) and Xcalibur 4.0 for peak area determination.

For testing PnpA activity against macromolecular PG, PGs from *Phdp* and several bacterial species, purified as described above, were incubated with 100 μg PnpA or inactive PnpA^C324A^ at 37°C overnight in 50 mM Tris (pH 8.0) and 300 mM NaCl. PGs incubated with vehicle were used as controls. After digestion, PGs were analyzed by HPLC or HPLC/HRMS as described above.

### Accession number(s).

The draft genome sequence of strain MT1415 was obtained and deposited in the GenBank database under accession number SUMH00000000. The experimental data were deposited with the Structural Biology Data Grid (73) under accession number https://doi.org/10.15785/SBGRID/736. The structure factors and atomic coordinates of PnpA are deposited in the RCSB Protein Data Bank with accession number 6SQX.
