# Supplementary figures and images for: A Secreted NlpC/P60 Endopeptidase from Photobacterium damselae subsp. piscicida Cleaves the Peptidoglycan of Potentially Competing Bacteria
Source: mSphere. 2021 Feb 3;6(1):e00736-20. doi: 10.1128/mSphere.00736-20 (PMC7860986; doi:10.1128/mSphere.00736-20)

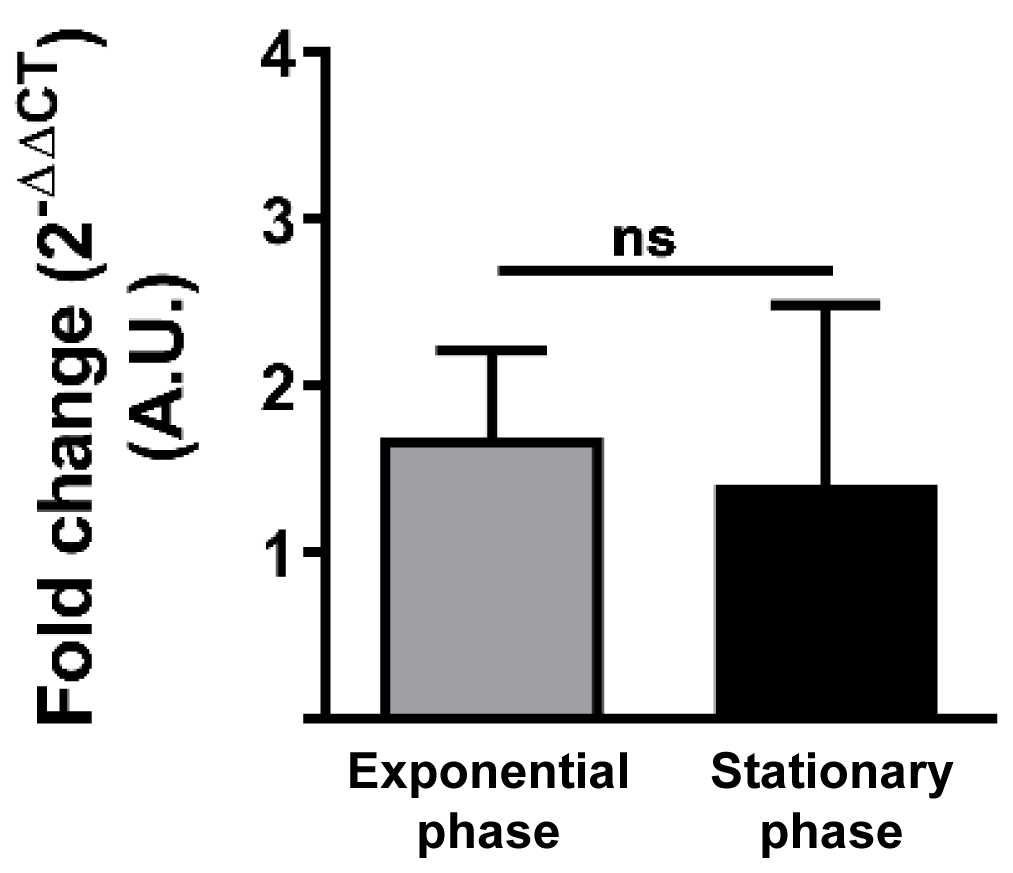

Supplement: FIG S1 [file mSphere.00736-20-sf001.tif]

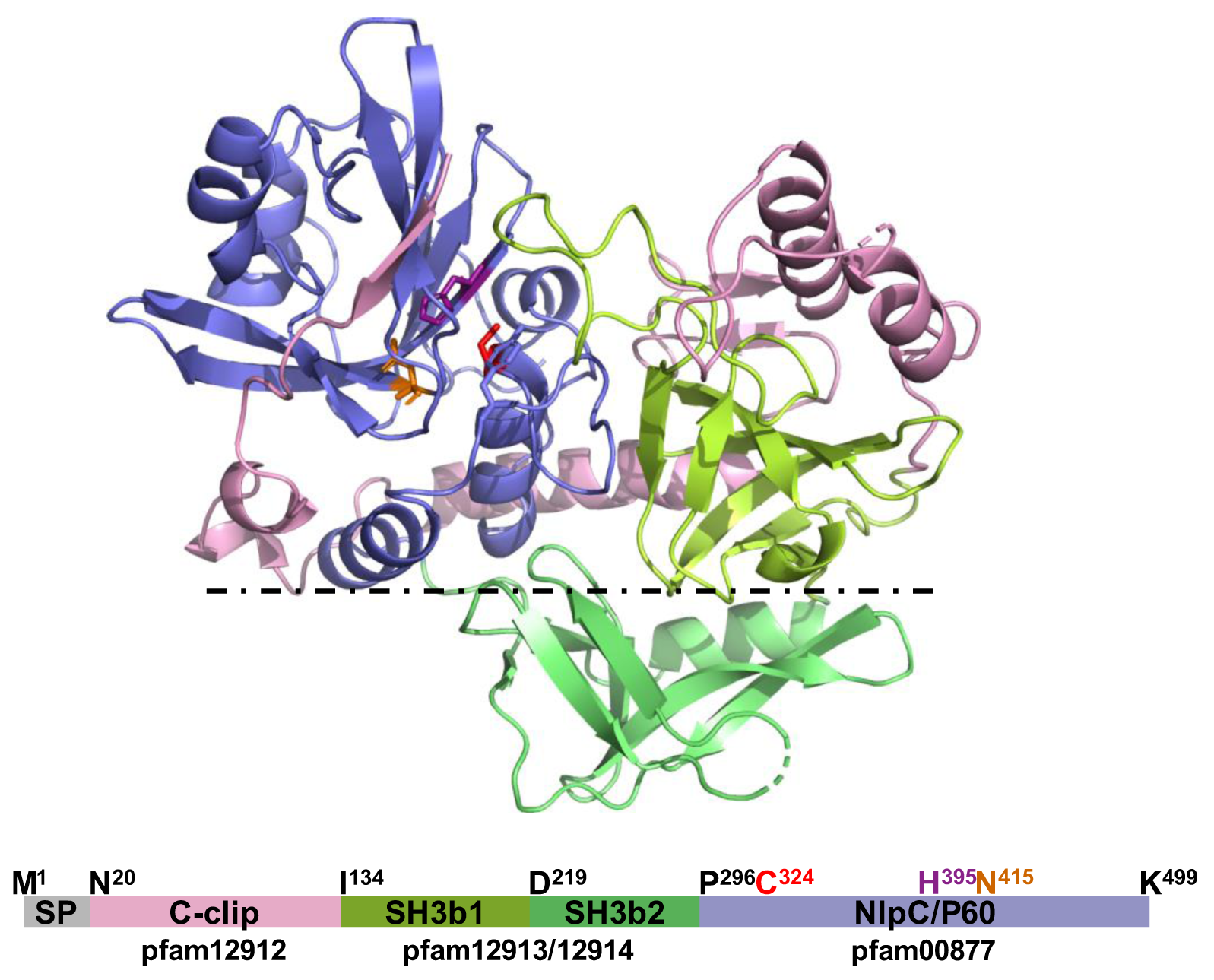

Supplement: FIG S2 [file mSphere.00736-20-sf002.tif]

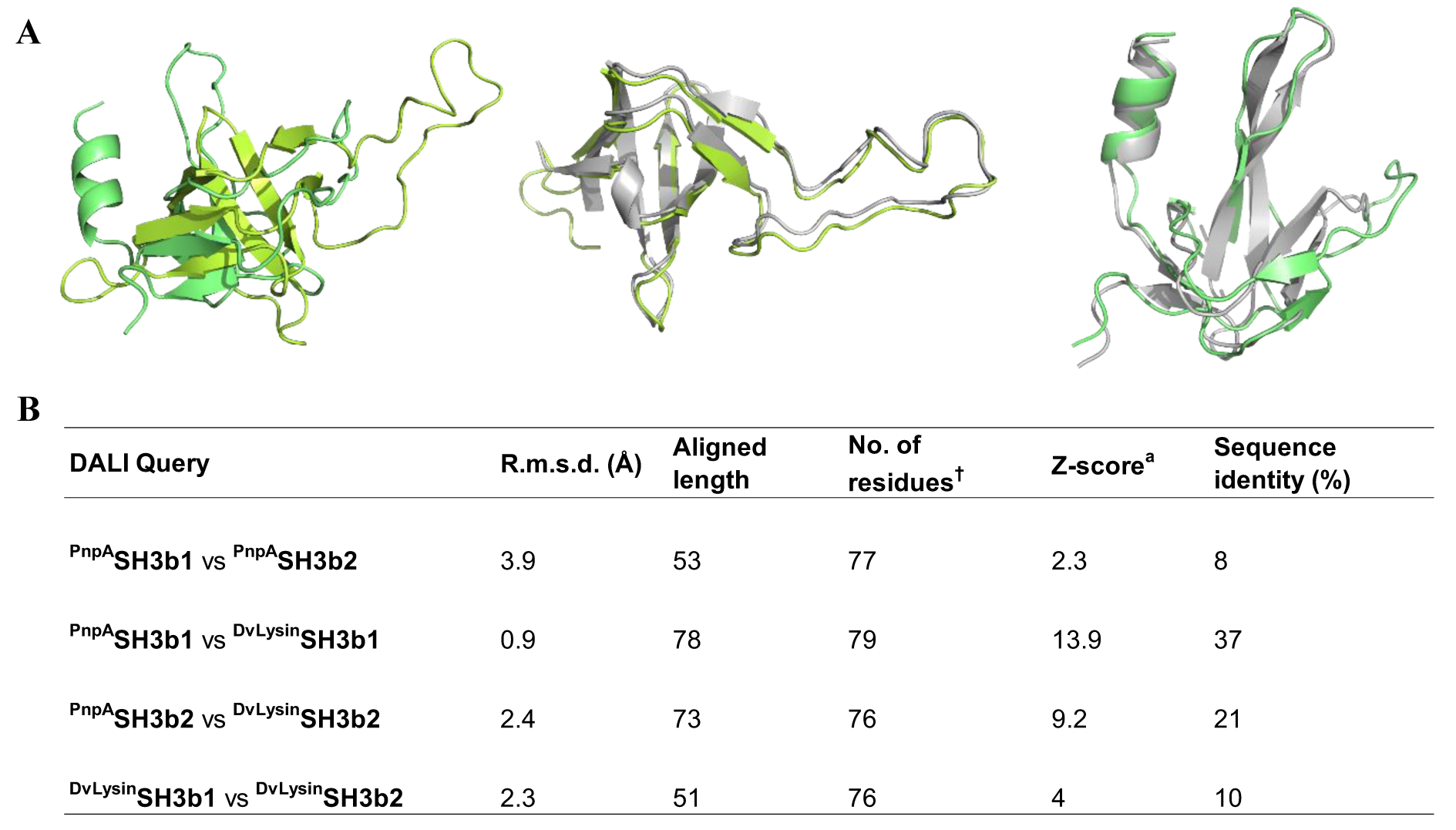

Supplement: FIG S3 [file mSphere.00736-20-sf003.tif]

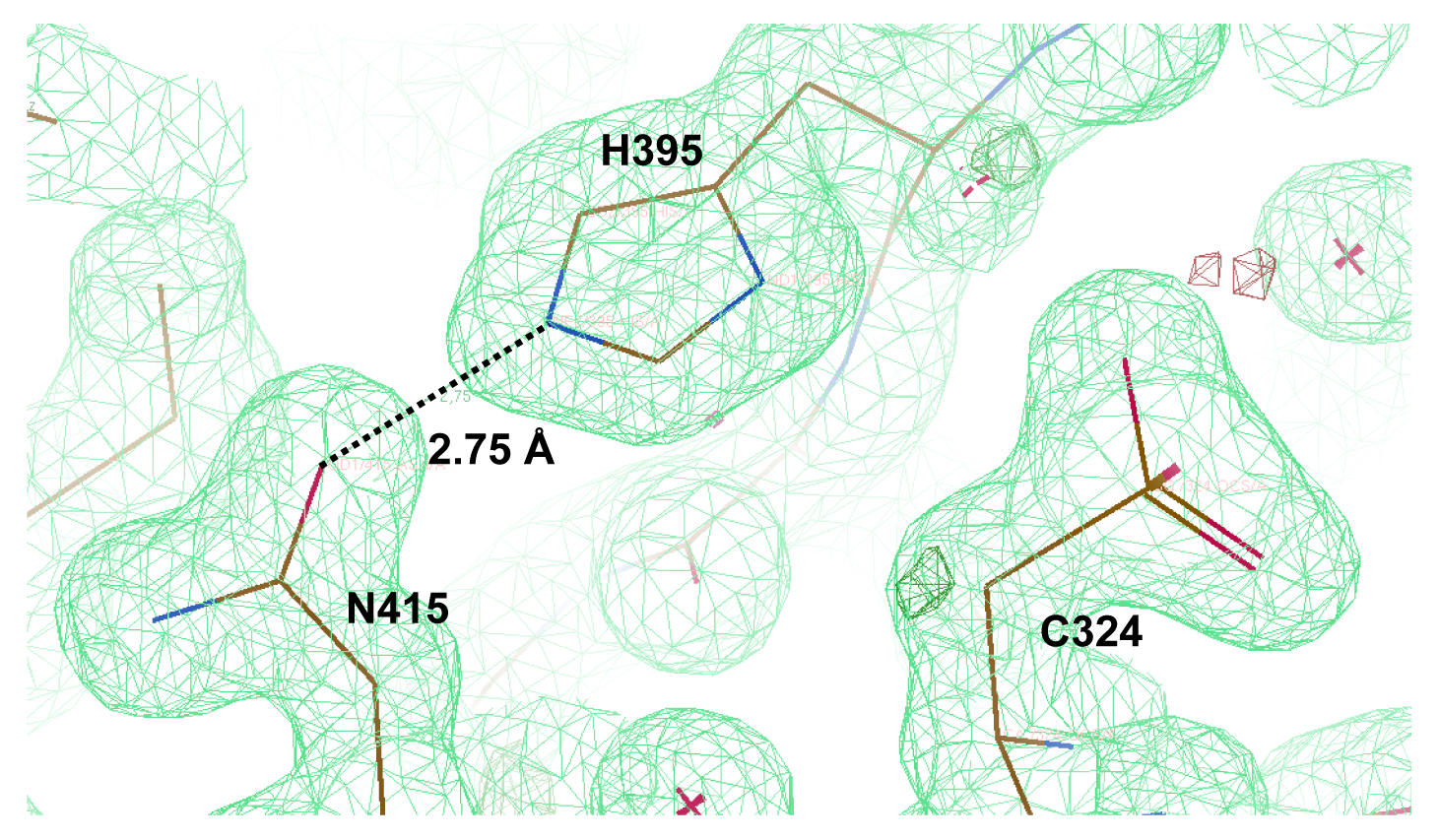

Supplement: FIG S4 [file mSphere.00736-20-sf004.tif]

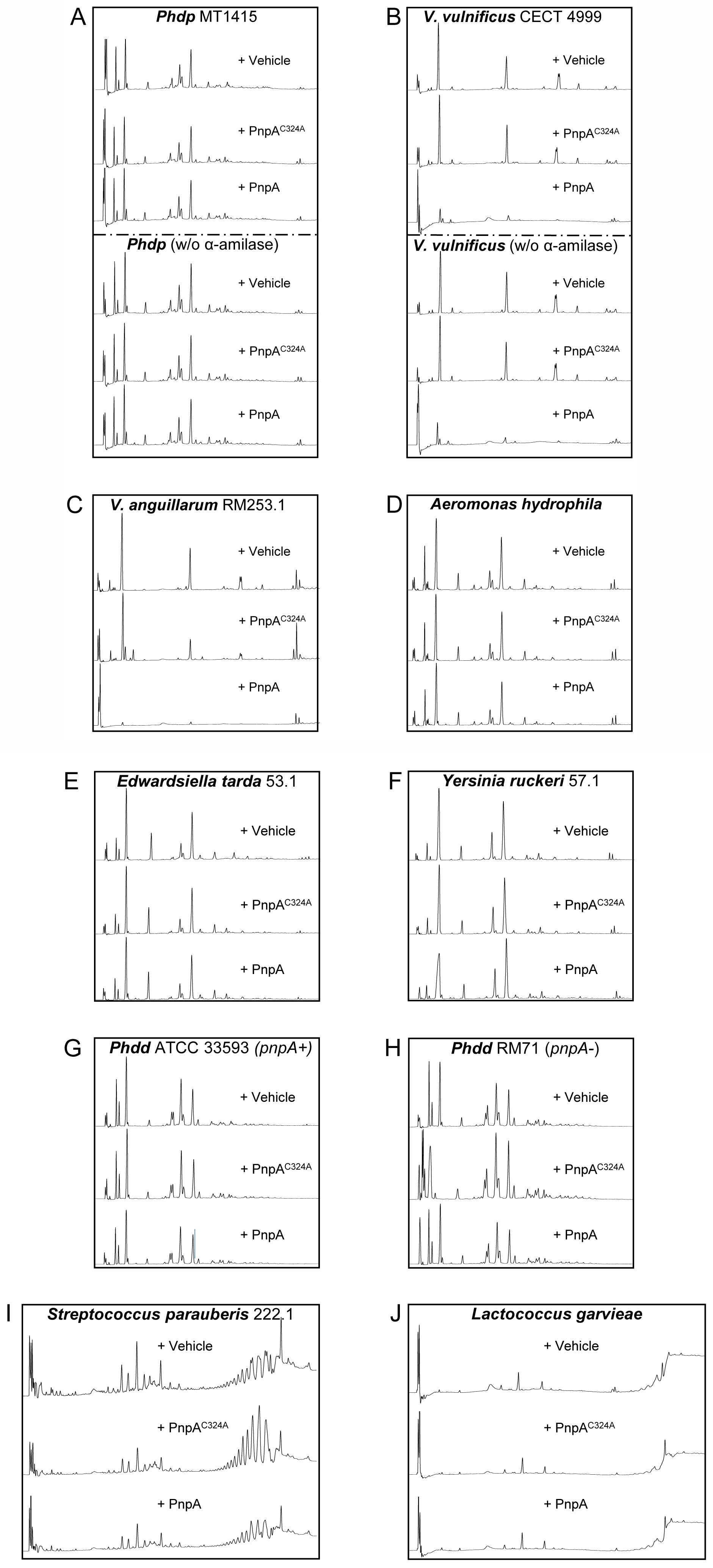

Supplement: FIG S5 [file mSphere.00736-20-sf005.tif]

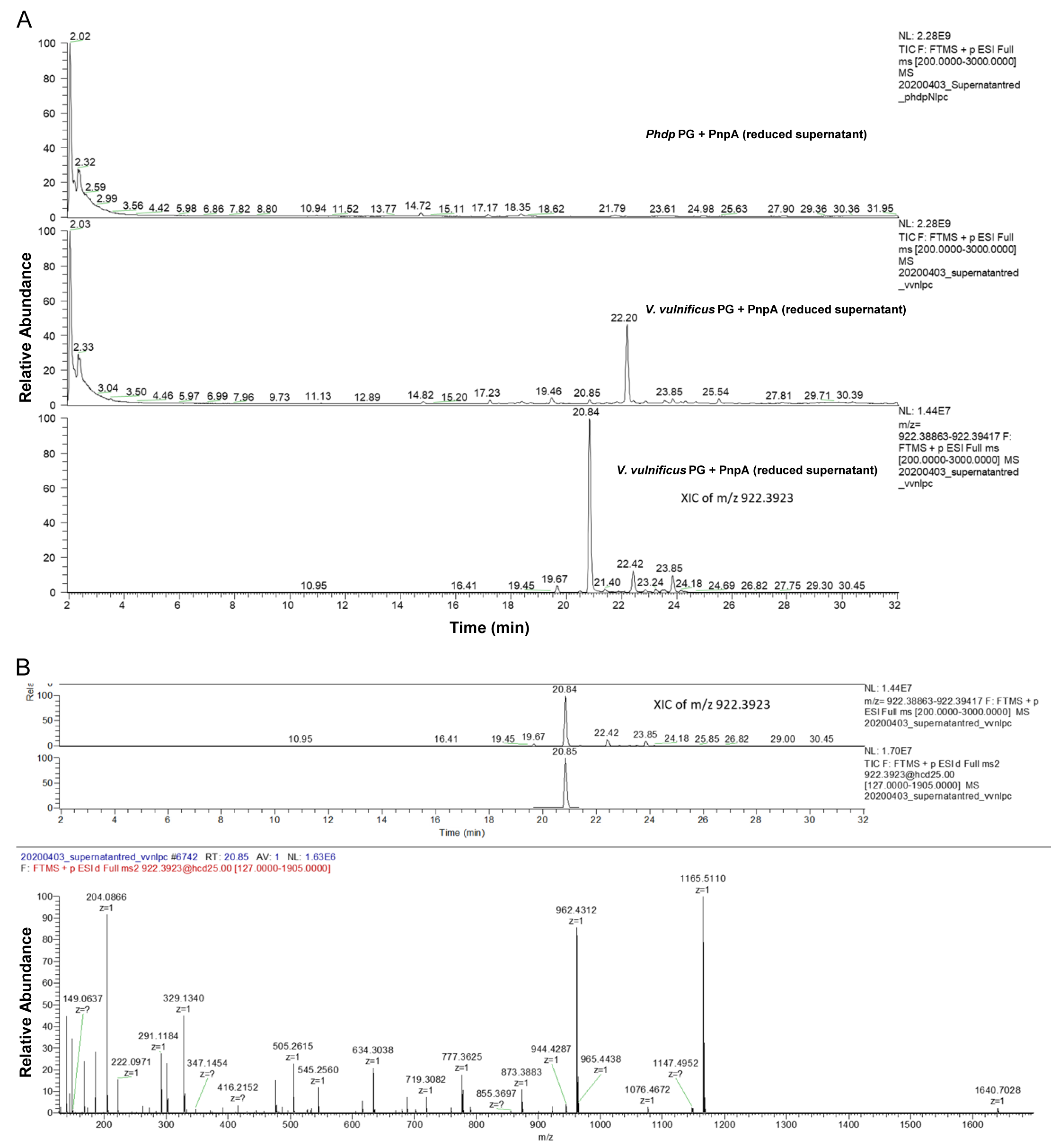

Supplement: FIG S6 [file mSphere.00736-20-sf006.tif]

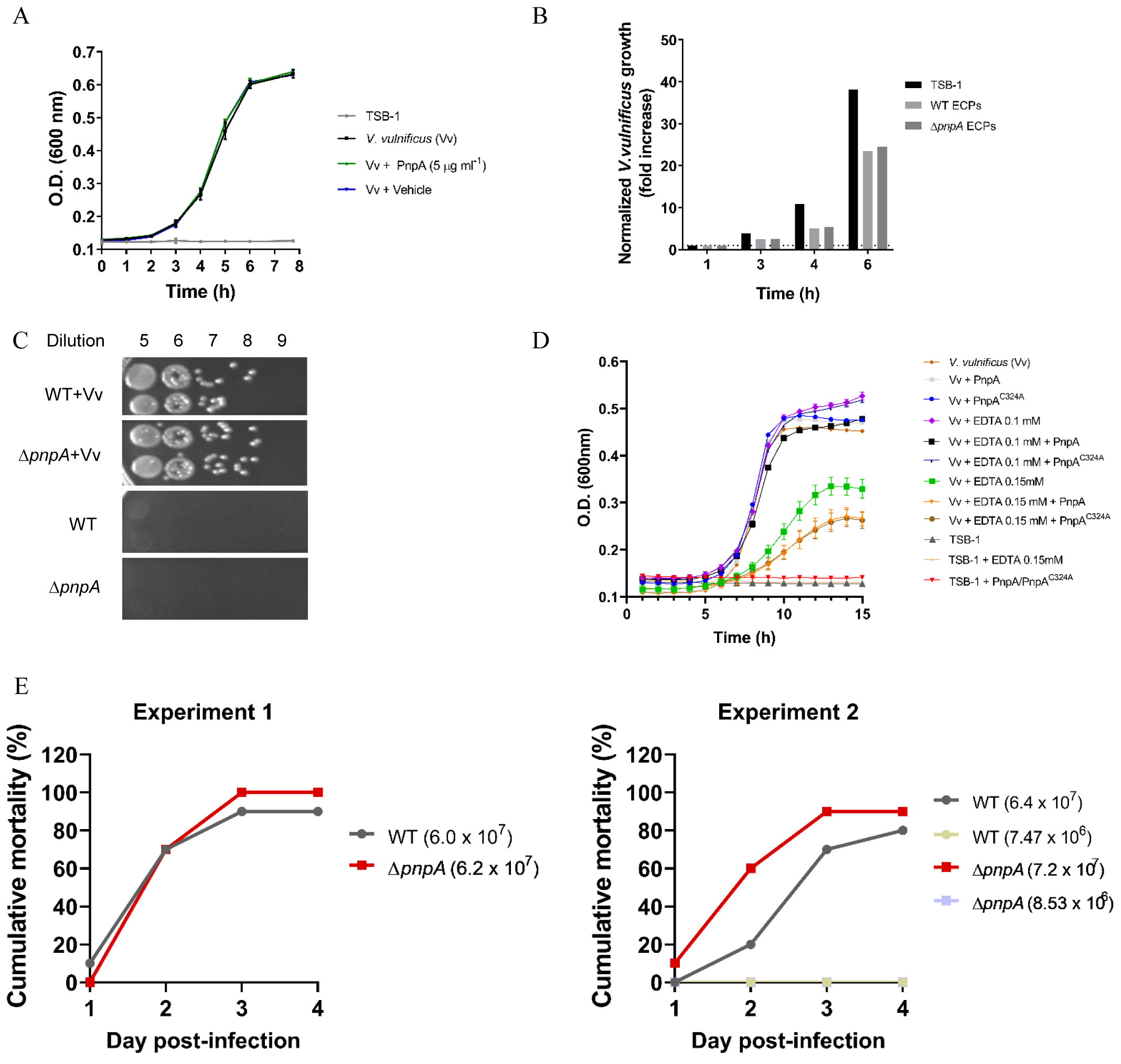

Supplement: FIG S7 [file mSphere.00736-20-sf007.tif]

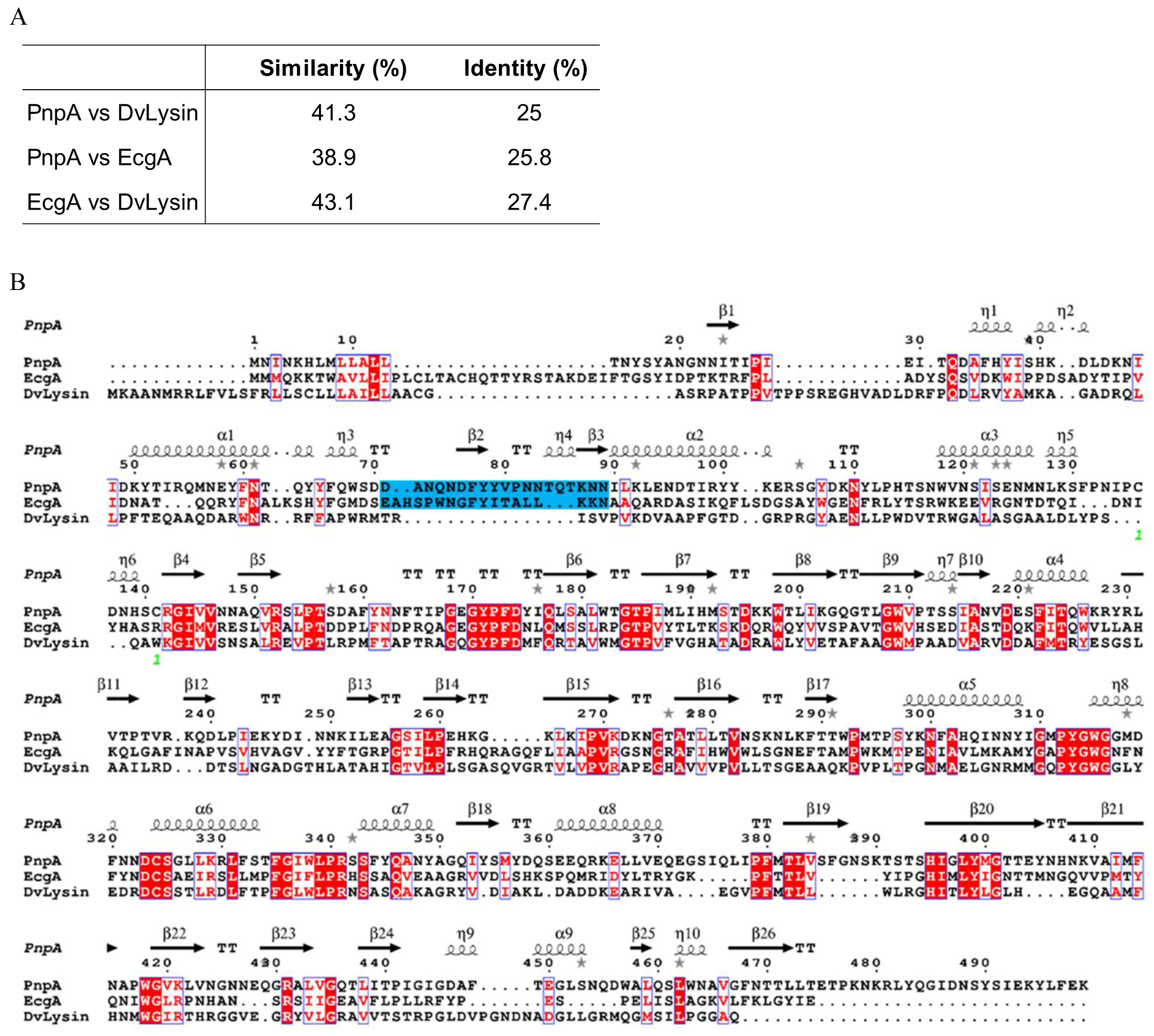

Supplement: FIG S8 [file mSphere.00736-20-sf008.tif]
