# Supplementary material for: A Secreted NlpC/P60 Endopeptidase from Photobacterium damselae subsp. piscicida Cleaves the Peptidoglycan of Potentially Competing Bacteria
Source: mSphere. 2021 Feb 3;6(1):e00736-20. doi: 10.1128/mSphere.00736-20 (PMC7860986; doi:10.1128/mSphere.00736-20)
Supplement: TABLE S1 [file mSphere.00736-20-st001.pdf]

	PnpA
<u>Data Collection</u>	
Space Group	$P2_12_12_1$
Wavelength (Å)	0.9786
Cell dimensions	
a,b,c (Å)	76.6, 110.0, 130.9
Resolution (Å)	38.3 - 1.40 (1.45 - 1.40)
Number of observations measured	1,194,493 (115,963)
Number of unique reflections measured	216,685 (21,426)
Multiplicity	5.5 (5.4)
Completeness (%)	99.8 (99.4)
I/σI	18.9 (0.85)
Wilson B-factor	20
R_{merge}	0.046 (1.47)
CC (1/2) (%)	100 (61.6)
Monomers per asymmetric unit	2
Matthews coefficient ($Å^3$ Da^{-1})	2.45
Solvent content (%)	49.9
<u>Refinement</u>	
R_{work}/R_{free} (%)	14.7 / 17.5
Numbers of non-hydrogen atoms	8870
macromolecules	7646
ligands	169
water	1055
Protein residues	914
RMSD from standard stereochemistry	
Bond lengths (Å)	0.011
Bond angles (º)	1.08
Ramachandran plot statistics	
Favored (%)	98.5
Allowed (%)	1.4
Disallowed (%)	0.1
**PDB Code**	**6SQX**
