# Supplementary material for: A Secreted NlpC/P60 Endopeptidase from Photobacterium damselae subsp. piscicida Cleaves the Peptidoglycan of Potentially Competing Bacteria
Source: mSphere. 2021 Feb 3;6(1):e00736-20. doi: 10.1128/mSphere.00736-20 (PMC7860986; doi:10.1128/mSphere.00736-20)
Supplement: TABLE S2 [file mSphere.00736-20-st002.pdf]

Aligned substructure(s)	PDB code	Reference	R.m.s.d. (Å)	Aligned length	No. of residues[†]	Z-score[a]	Sequence identity (%)	Comments
**C-clip + SH3b + NlpC/P60**	6SQX	This study						putative gamma-D-glutamyl-L-diamino acid endopeptidase PnpA
	3M1U	JCSG[‡]	2.2	407	420	43.3	26	putative gamma-D-glutamyl-L-diamino acid endopeptidase DvLysin
**SH3b + NlpC/P60**	3H41	(1)	2.6	251	307	22.4	19	gamma-D-glutamyl-L-diamino acid endopeptidase YkfC
	3NPF	JCSG[‡]	2.9	256	305	21.2	19	putative dipeptidyl-peptidase VI
	4R0K	JCSG[‡]	3	253	304	21.1	20	putative dipeptidyl-peptidase VI
	3PVQ	JCSG[‡]	2.8	246	297	20.5	20	putative dipeptidyl-peptidase VI
	2HBW	(2)	2.4	182	220	14	19	putative endopeptidase AvPCP
	2EVR	(2)	2.4	182	222	13.9	19	putative gamma-d-glutamyl-l-diamino acid endopeptidase NpPCP
	6BIQ	(3)	4.7	159	266	10.9	16	NlpC/P60 D,L endopeptidase (NlpC_A2)
	6BIO	(3)	4.5	159	278	10.7	15	NlpC/P60 D,L endopeptidase (NlpC_A1)
**Lysozyme-Like + NlpC/P60**	4FDY	(4)	2.3	115	295	11.5	21	bifunctional Cell Wall Hydrolase CwlT
	4HPE	JCSG[‡]	2.7	121	290	11.3	22	putative cell wall hydrolase
**Coil-Coil Domain + NlpC/P60**	6B8C	(5)	2.3	108	117	10.7	23	NlpC/p60 domain of peptidoglycan hydrolase SagA
**LysM + NlpC/P60**	4XCM	(6)	2.7	115	218	11.2	19	putative NlpC/P60 D,L endopeptidase LysM
**NlpC/P60**	3I86	Unpublished	2.4	117	136	11.1	26	P60 Domain
	2K1G	(7)	2.2	115	129	10.9	24	NlpC/P60 domain of lipoprotein Spr
	3GT2	Unpublished	2.3	114	135	10.4	21	P60 Domain
	4JXB	(8)	2.5	113	130	10.2	22	RipD, a non-catalytic NlpC/p60 domain protein
	3PBI	(9)	2.6	126	199	9.4	25	peptidoglycan hydrolase RipB
	3NE0	(10)	4.1	130	208	8.4	24	RipA, a Mycobacterial Enzyme
	2XIV	Unpublished	4.1	129	207	8.3	23	Rv1477, Hypothetical Invasion Protein
	3PBC	(9)	4.2	129	208	8.3	25	Peptidase module of the peptidoglycan hydrolase RipA
	4Q4N	(11)	4.5	131	208	8.3	22	RipA, a Mycobacterial Enzyme
	4EYZ	(12)	3.3	140	246	7.8	15	Cellulosome-related protein module
	6IST	Unpublished	3.4	119	213	7.5	8	Endolysin LysIME-EF1

References

1. Xu, Q. et al. Acta Crystallographica Section F 2010, 66:1354-1364.
2. Xu, Q. et al. Structure 2009, 17:303-313.
3. Pinheiro, J. et al. Mbio 2018, 9.
4. Xu, Q. et al. Journal of Molecular Biology 2014, 426:169-184.
5. Kim, B. et al. eLife 2019, 8:e45343.
6. Wong, J.E.M.M. et al. Acta Crystallographica Section D 2015, 71:592-605.
7. Aramini, J.M. et al. Biochemistry 2008, 47:9715-7.
8. Böth, D. et al. Biochemical Journal 2014, 457:33-41.
9. Both, D. et al. J Mol Biol 2011, 413:247-60.
10. Ruggiero, A. et al. Structure 2010, 18:1184-90.
11. Squeglia, F. et al. Acta Crystallogr D Biol Crystallogr 2014, 70:2295-300.
12. Levy-Assaraf, M. PLOS ONE 2013, 8:e56138.
